# Interpretable machine learning for early prediction of sepsis-induced coagulopathy: a multicenter retrospective development and validation study

**DOI:** 10.1186/s12911-026-03471-8

**Published:** 2026-04-14

**Authors:** Qingyun Peng, Yanzi Guo, Haoyuan Tang, Shuai Liu, Wei Huang, Xinlong Chen, Shijia Zhong, Zeyuan Zhao, Haofei Wang, Wenhan Hu, Shuhe Yang, Jianfeng Xie, Ming Xue, Shuyuan Qian, Xiaojing Wu, Yingzi Huang

**Affiliations:** 1 Jiangsu Provincial Key Laboratory of Critical Care Medicine, Department of Critical Care Medicine, School of Medicine, Zhongda Hospital, Southeast University, No. 87, Dingjiaqiao Road, Gulou District, Nanjing, 210009 People’s Republic of China; 2 Department of Critical Care Medicine, Affiliated Hospital of Nantong University, Nantong, Jiangsu 226001 China

**Keywords:** Sepsis induced coagulopathy, Machine learning, Early prediction, XGBoost model, SHAP interpretability

## Abstract

**Background:**

Sepsis-induced coagulopathy (SIC) is a frequent complication of sepsis that significantly impacts clinical outcomes, underscoring the necessity for early identification to facilitate timely intervention. This study aimed to develop an interpretable machine-learning model to predict early onset SIC within 3 days.

**Methods:**

In this multicenter retrospective cohort study, four distinct cohorts derived from the MIMIC-IV dataset, the eICU Collaborative Research Database, and an ICU cohort from Zhongda Hospital, Southeast University, were used for model derivation and validation. Core predictive variables were identified through multi-step feature engineering using data collected within the first 24 h of ICU admission. Five machine learning models were trained based on these features and subsequently evaluated on three external validation cohorts.

**Results:**

The derivation cohort, drawn from MIMIC-IV v2.2 database, included 11,033 patients. External validation encompassed three cohorts totaling 5,572 patients: MIMIC-IV v3.1 dataset (*n* = 1,371), eICU Database (*n* = 2,848), and Zhongda Hospital database (*n* = 1,353). Among five evaluated machine learning models, XGBoost demonstrated the best discriminative performance. The final model incorporated seven predictive variables: International Normalized Ratio, lactate, platelet count, Sequential Organ Failure Assessment (SOFA) circulation, renal, and respiratory subscores, and mechanical ventilation status. The model achieved robust prediction of early-stage SIC across validation sets (internal validation set AUC = 0.83; internal test set AUC = 0.82; external validation set AUC = 0.93,0.86,0.83, respectively). However, the model was ineffective for predicting late-onset SIC, with an internal test set AUC of 0.5 and external validation set AUCs of 0.48, 0.53, and 0.6, respectively.

**Conclusion:**

This study establishes the feasibility of an interpretable XGBoost model for accurately predicting early-onset SIC. As a promising clinical decision-support tool, its real-world utility and impact on patient outcomes should be further established through prospective validation prior to deployment.

**Supplementary Information:**

The online version contains supplementary material available at 10.1186/s12911-026-03471-8.

## Introduction

Sepsis is a life-threatening organ dysfunction caused by a dysregulated host response to infection [1]. The excessive release of inflammatory mediators and activation of the coagulation system play crucial roles in the pathogenesis of sepsis [2]. Activated leukocytes and platelets, together with endothelial injury, are central drivers of thromboinflammation in sepsis, leading to widespread microthrombosis in capillaries that impairs tissue perfusion and ultimately contributes to organ failure [3, 4]. Patients typically exhibit coagulation abnormalities, progressing to sepsis-induced coagulopathy (SIC) [5].

Clinical studies demonstrate that SIC occurs in 22%–60% of patients with sepsis, with an observed mortality ranging from 26% to 53% [6–9]. Furthermore, 20%-50% of these SIC patients progress to disseminated intravascular coagulation (DIC) despite supportive care [6, 9]. The mortality among DIC patients ranges from 31% to 86%, markedly exceeding that of non-DIC patients and indicating a substantial worsening of clinical outcomes [10, 11].

Early detection of coagulation disorders is crucial for assessing the severity and predicting the prognosis [12]. SIC is regarded as an early phase of DIC because it includes most cases of overt DIC [13]. Notably, most SIC cases either manifest at sepsis diagnosis or develop within the initial four-day window post-diagnosis [6].

Although the International Society on Thrombosis and Haemostasis (ISTH) has established diagnostic criteria for SIC, which incorporate platelet count (PLT), International Normalized Ratio (INR), and Sequential Organ Failure Assessment (SOFA) score to standardize clinical identification [7], a validated tool for predicting SIC onset is still lacking. This limitation arises because the International Society on Thrombosis and Haemostasis sepsis-induced coagulopathy (ISTH-SIC) score is designed for diagnosis rather than prediction [14]. For instance, previous studies demonstrated that this score yielded high sensitivity but low specificity for early risk identification [8, 14, 15]. Furthermore, it provides only a static snapshot and fails to effectively capture the rapid dynamic trends that are most predictive [16]. As a result, the SIC score is more adept at confirming the condition than predicting it, highlighting the urgent need for multivariate predictive models.

Machine learning (ML) has shown potential in critical care medicine, with various studies suggesting its utility in predicting disease progression and patient outcomes through the analysis of large-scale, multidimensional clinical datasets [17–20]. Advanced ML models are particularly adept at capturing higher-order interactions between covariates and clinical outcomes, thereby excelling in extracting actionable insights from complex, data-intensive environments [21, 22]. Emerging evidence demonstrates their capacity for early and temporally sensitive prediction of SIC through continuous analysis of heterogeneous medical data streams [23, 24]. Numerous ML techniques have been applied in developing SIC prediction models, many of which exhibit promising performance [23–26]. However, the “black-box” nature of many ML algorithms limits their interpretability and clinical adoption [27]. There remains a critical unmet need for clinically actionable predictive models for SIC in contemporary critical care practice.

This study aims to develop and validate an interpretable and clinically applicable ML model to predict both early-onset (within 3 days) and late-onset (4 to 7 days) SIC, with decision-critical feature visualization using Shapley Additive Explanations (SHAP) values.

## Methods

### Data source and study population

This multicenter retrospective cohort study utilized data from three independent sources: The Medical Information Mart for Intensive Care IV database (MIMIC-IV, versions 2.2 and 3.1; https://mimic.mit.edu), the eICU Collaborative Research Database (eICU-CRD, version 2.0; https://physionet.org), and the Intensive Care Unit (ICU) of Zhongda Hospital, Southeast University. The dataset was stratified into two mutually exclusive cohorts: (1) Derivation cohort: Data extracted from the MIMIC-IV database (version 2.2, 2008–2019), with disease screening and data collection performed using both the International Classification of Diseases (ICD)-9 and ICD-10 coding systems; (2) External validation cohort: This cohort was further divided into three subgroups: Cohort 1: Data from the MIMIC-IV database (version 3.1, data extracted from 2020 to 2022); Cohort 2: Data sourced from the eICU Database (2014–2015); Cohort 3: Data derived from a septic patient database comprising individuals admitted to the ICU of Zhongda Hospital (from November 2016 to November 2023).

Authorization required completion of the Collaborative Institutional Training Initiative (CITI) program by the US National Institutes of Health. *Zhao* and *Zhong* completed the online examination and obtained certification numbers (Record IDs: 14568635,12073220). This retrospective study was conducted in accordance with the Declaration of Helsinki (1964) and its later amendments. Ethical approval was waived for MIMIC-IV and eICU analyses as it contains publicly available anonymized data. For the Zhongda Hospital sepsis database, approval was granted by the Institutional Review Board of Zhongda Hospital, Southeast University (Approval No. 2025ZDSYLL253-P01), with informed consent waived due to the retrospective observational design.

Patient eligibility required meeting all the following inclusion criteria: (1) age ≥ 18 years, (2) ICU stay > 48 h, and (3) diagnosis of sepsis based on the Sepsis-3 criteria [1]. The derivation cohort excluded patients with traumatic or hemorrhagic shock, hematologic or solid malignancies, acute/subacute hepatic necrosis or cirrhosis, and pregnancy. In contrast, the external validation cohorts intentionally retained these patients to evaluate model performance and generalizability in a broader, real-world population. Our study adheres to the Transparent Reporting of a multivariable prediction model for Individual Prognosis or Diagnosis (TRIPOD) guideline [28–30](detailed in Supplementary File 2). Additionally, in accordance with reporting standards for retrospective observational studies, the STROBE (Strengthening the Reporting of Observational Studies in Epidemiology) checklist is provided in Supplementary File 3 [31].

### Sepsis and sepsis-induced coagulopathy definition

According to the Sepsis-3 criteria, sepsis was defined as a suspected or confirmed infection combined with an acute increase in SOFA score of ≥ 2 points [1]. In this study, we operationalized sepsis based on established methodologies (Supplementary method A in Supplementary File 1) [32–34]. SIC was diagnosed according to the ISTH-SIC criteria, with patients who met the diagnostic threshold (a composite score ≥ 4) identified as SIC cases [7]. SIC onset was stratified into an early phase (≤ 72 h after ICU admission) and a late phase (occurring between days 4 and 7).

### Data collection and preprocessing

To predict SIC, we collected a total of 51 clinical variables from septic patients within 24 h of ICU admission (Table S1). The variables included: Demographic data (age, sex, weight, height, BMI); SOFA scores with subsystem scores; Laboratory data (arterial blood gas, complete blood count, liver function, renal function, and coagulation profile); Vital signs (respiratory rate, blood pressure, heart rate, and temperature). Comorbidities (hypertension, diabetes mellitus, chronic kidney disease [CKD], and chronic liver disease) were also collected. Supportive treatments, such as heparin administration, mechanical ventilation (MV), and continuous renal replacement therapy (CRRT), were documented. Daily SOFA scores, INR, and platelet counts were recorded for seven consecutive days post-admission to calculate SIC scores. The worst daily values of these parameters were systematically extracted from electronic health records. The bilirubin concentration is the variable most frequently missing from the SOFA score [35, 36], typically because clinicians assume the level is normal and thus omit its measurement. Consequently, in the calculation of the score, missing values are treated as normal, corresponding to a SOFA sub-score of 0. Clinical outcomes, including ICU and hospital length of stay, 28-day mortality, ICU mortality, and hospital mortality, were recorded. The machine learning task was formulated as a binary classification problem to predict whether patients would develop SIC between day 2 and day 7 following sepsis diagnosis.

Prior to model development, comprehensive data cleansing was performed on both the derivation and external validation cohorts, with 51 features harmonized across all datasets. To prevent data leakage, the derivation cohort was first stratified into a training set (70%), an internal validation set (20%), and an internal test set (10%) before any imputation [37]. Variables with missing values exceeding 35% were excluded, resulting in the removal of 13 variables in total. The missingness mechanism for remaining variables was assessed using Little’s Missing Completely at Random (MCAR) test; if rejected, logistic regression likelihood ratio tests (LRTs) were used to distinguish between Missing at Random (MAR) and Missing Not at Random (MNAR). For variables with MAR mechanisms, missing values were imputed using a random forest algorithm (max_iter = 1000). The density distributions of features before and after imputation are shown in Figure S1, and the percentage of missing values for each variable is detailed in Table S2.

For dichotomous categorical variables, one-hot encoding was applied to convert them into a numerical format. To ensure optimal performance across different algorithms, tailored preprocessing strategies were employed: Tree-based models (Random Forest [RF], Extreme Gradient Boosting [XGBoost], and Light Gradient Boosting Machine [LightGBM]) utilized the raw or one-hot encoded feature values to preserve their intrinsic decision boundaries. In contrast, linear models (Logistic Regression [LR] and Support Vector Machine [SVM]) were trained on z-score normalized data to ensure feature comparability and stabilize the convergence process.

### Feature engineering

Feature engineering was performed to optimize model performance through a four-step sequential selection of clinical variables. First, 38 variables were evaluated by five senior intensivists (> 10 years ICU experience). To reduce redundancy and multicollinearity, the total SOFA score, its coagulation subscore (based solely on platelet count), and the neurological subscore (excluded due to subjectivity and susceptibility to sedative effects) were excluded. Thirty-five clinically relevant and interpretable variables were retained, which subsequently underwent missing value handling, imputation, and standardization as previously described. Second, four distinct feature sets were generated using complementary filter and wrapper methods: Kruskal-Wallis testing for non-parametric Analysis of Variance (ANOVA); 5-fold Recursive Feature Elimination with Cross-Validation (RFECV) using Random Forest and Logistic Regression as base learners, which automatically identifies optimal features through iterative elimination of low-importance variables; and Null Importance screening via randomized permutation testing to detect robust features through information gain and tree splitting [38]. This yielded 36 features from Kruskal-Wallis (including one-hot encoded categorical states), 33 from RFECV-RF, 35 from RFECV-LR, and 42 from Null Importance screening. The top 30% highest-ranked features from each method were aggregated into a candidate feature set [39]; these features and their importance weights are presented in Figure S2. Third, these sets were evaluated for stability (quantified via Population Stability Index) and efficacy (measured by cross-validated the area under the receiver operating characteristic curve [AUROC]), with final selection determined by a composite score weighting stability 60% and efficacy 40% [40, 41]. The 13 Null Importance-derived features achieved the highest composite score (Table S3). Fourth, collinearity was assessed using Pearson’s correlation dendrograms (Figure S3). Subsequently, the senior intensivists evaluated the feature subset by applying three criteria: clinical relevance to coagulation disorders, immediate bedside availability, and practical interpretability. Ultimately, seven variables were selected for the development of the SIC prediction model. Density distribution plots of these selected features across the training and external validation sets are shown in Figure S4.

### Model development and performance evaluation

ML models were developed using the final feature set. Our model enforces a strict temporal separation between the predictor window and the outcome window [30]. Specifically, all predictors were extracted exclusively from the initial 24 h (Day 1) following ICU admission, while the outcome (SIC occurrence) was monitored during the subsequent early phase (Days 2 to 3) and late phase (Days 4 to 7). Five model types, including LightGBM, RF, XGBoost, LR, and SVM, were selected to model the categorical outcome variables. During the training process, a hybrid balancing strategy was employed, which involved generating synthetic samples using the Synthetic Minority Over-sampling Technique (SMOTE) algorithm and incorporating class weights into the loss function. This strategy was designed to enhance the model’s sensitivity toward the minority class and alleviate class imbalance caused by the skewed incidence of SIC in the external validation cohort. Additionally, probabilistic calibration was integrated into the model.

Hyperparameter optimization was conducted in two stages. First, a random search over key base parameters (e.g., max_depth values of 3, 6, 9, 12 for tree-based models; C values of 1e-2, 1e-1 for linear models) was performed on the internal validation set to identify candidate models. The candidate model achieving the optimal area under the curve (AUC) was then selected. Subsequently, Bayesian optimization was employed on the internal test set to further refine critical hyperparameters. Model interpretability was analyzed using the SHAP framework. Finally, the optimized model was evaluated on the external validation sets to assess its effectiveness and generalizability.

### Web deployment tool based on the streamlit framework

To facilitate the utility of the model in clinical settings, the final prediction model was implemented into a web application established based on the Streamlit Python-based framework. When the values of corresponding features from the final model are provided, the application can return the probability of SIC and the force plot for the individual patient.

### Statistical analysis

Statistical analyses were performed using Python 3.11.7 (via Jupyter Notebook 7.4.1) and R software version 4.3.3. The normality of continuous variables was assessed using the Kolmogorov-Smirnov tests and visual inspection of histograms and Q-Q plots. As all continuous clinical variables exhibited a non-normal distribution, they were reported as medians with interquartile ranges (IQRs) and compared using the Mann-Whitney U test. Categorical variables were expressed as frequencies and percentages (%) and analyzed using the chi-square test or Fisher’s exact test. Collinearity among variables was assessed, with a tolerance > 0.2 or a variance inflation factor (VIF) < 10 considered acceptable. After model development, predictive performance was rigorously evaluated using bootstrap-derived 95% confidence intervals based on 1,000 resampling iterations. Primary evaluation metrics included the AUROC and the area under the precision-recall curve (AUPRC). A comprehensive set of performance metrics, including the F1-score, accuracy, sensitivity, specificity, positive predictive value (PPV), and negative predictive value (NPV), was systematically calculated to characterize the model’s discriminatory capability across classification thresholds. To rigorously validate the robustness of the Early-SIC model and ensure its clinical reliability across heterogeneous populations, we performed multi-dimensional sensitivity analyses covering: (i) impact of baseline comorbidities, (ii) data missingness, (iii) clinical interventions, and (iv) lead-time analysis (as detailed in Supplementary Method B in Supplementary File 1). The optimal cutoff point was determined by maximizing the Youden index, calculated as (sensitivity + specificity − 1). A two-sided p-value < 0.05 was considered statistically significant.

## Results

### Patient characteristics

**Fig. 1:**
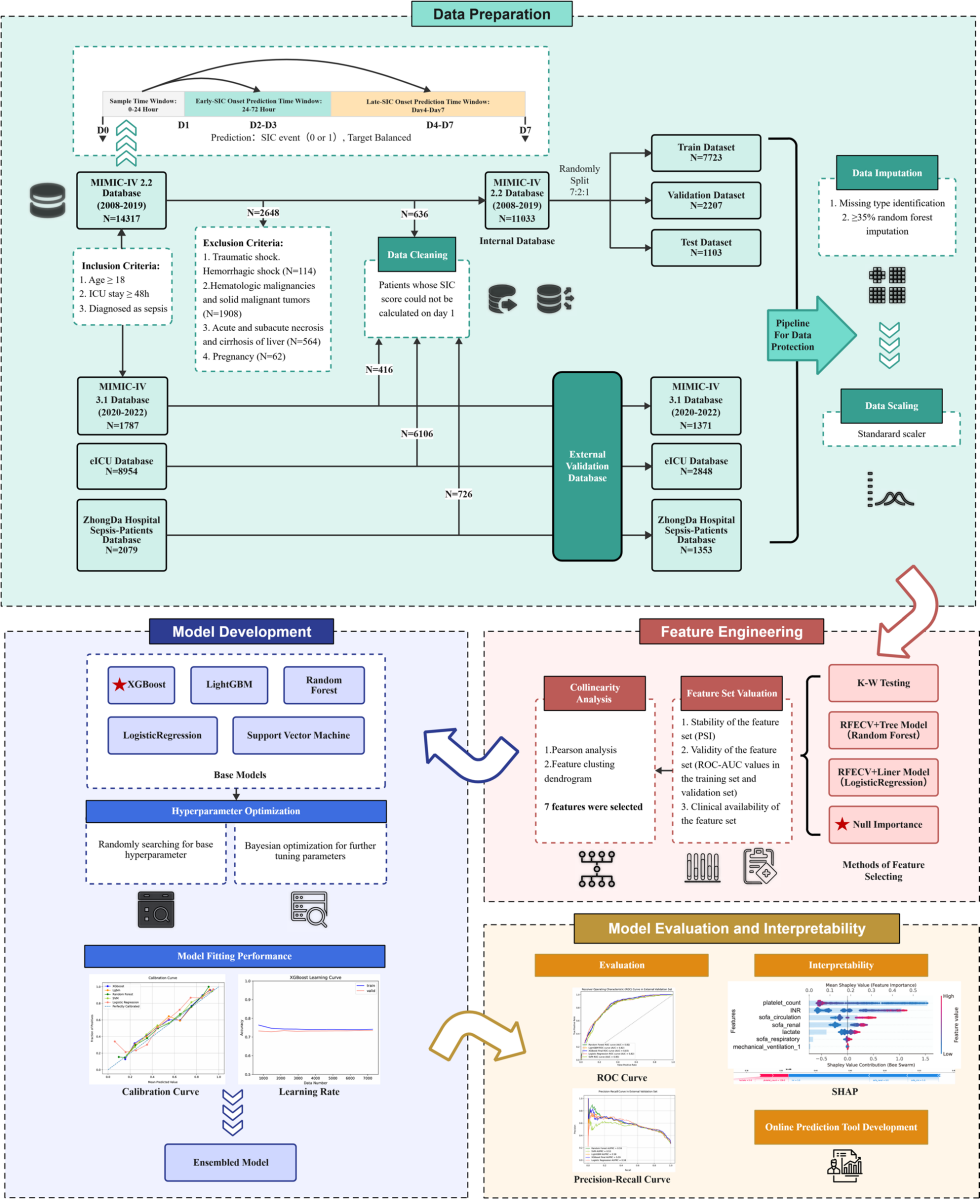
Flow chart of the study design. SIC: Sepsis induced coagulopathy; MIMIC-IV: Medical Information Mart for Intensive Care IV; ICU: Intensive Care Unit; eICU: eICU Collaborative Research Database; K-W: Kruskal-Wallis; RFECV: Recursive Feature Elimination with Cross-Validation; PSI: Population Stability Index; ROC: Receiver Operating Characteristic; AUC: Area Under Curve; XGBoost: Extreme Gradient Boosting; LightGBM: Light Gradient Boosting Machine; SVM: Support Vector Machine; SHAP: SHapley Additive explanation; INR: International Normalized Ratio; SOFA: Sequential Organ Failure Assessment

This multicenter retrospective study initially included 27,137 patients with sepsis from three medical centers. After applying predefined exclusion criteria and data cleaning for critical missing entries or duplicates, the final study population comprised 16,605 patients (Fig. 1). This included a derivation cohort from MIMIC-IV v2.2 (*n* = 11,033) and three external validation cohorts: MIMIC-IV v3.1 (Cohort 1, *n* = 1,371), eICU (Cohort 2, *n* = 2,848), and Zhongda Hospital (Cohort 3, *n* = 1,353), which collectively contained 5,572 patients. Demographic and clinical characteristics of the derivation and external validation cohorts are compared in Table 1.

**Table 1:** Clinical characteristics between the derivation cohort and external validation cohorts

Cohort Variables	Derivation cohort (MIMIC-IV 2.2) *n* = 11,033	External validation cohort (MIMIC-IV 3.1) *n* = 1371	*p*-value*	External validation cohort (eICU) *n* = 2848	*p*-value*	External validation cohort (Zhongda Hospital) *n* = 1353	*p*-value*
**Demographics**							
Age (years)	68 (56, 79)	66 (55, 76)	< 0.001	66 (56, 77)	< 0.001	69 (56, 78)	0.763
Gender (Male, %)	6321 (57.3)	538 (39.2)	< 0.001	1518 (53.3)	0.002	909 (67.2)	< 0.001
Weight (kg)	80 (67, 95)	82 (70, 98)	< 0.001	81 (66, 99)	0.034	65 (60, 70)	< 0.001
Height (cm)	170 (163, 178)	170 (163, 178)	0.346	170 (162, 178)	0.298	170 (160, 172)	< 0.001
BMI (kg/m²)	27.8 (24.1, 32.7)	28.7 (24.5, 34)	< 0.001	27.9 (23.3, 33.8)	0.682	25.6 (21.2, 30.6)	< 0.001
**Disease severity scores (Median (IQR))**							
SOFA	6 (4, 8)	7 (5, 10)	< 0.001	8 (6, 11)	< 0.001	8 (6, 11)	< 0.001
SOFA of respiratory	3 (0, 3)	3 (2, 4)	< 0.001	3 (1, 4)	< 0.001	2 (0, 3)	< 0.001
SOFA of coagulation	0 (0, 1)	0 (0, 1)	0.003	0 (0, 2)	< 0.001	1 (0, 2)	< 0.001
SOFA of liver	0 (0, 1)	0 (0, 0)	< 0.001	0 (0, 1)	0.004	0 (0, 1)	0.789
SOFA of circulation	1 (1, 3)	1 (1, 4)	0.026	1 (1, 1)	< 0.001	4 (3, 4)	< 0.001
SOFA of CNS	0 (0, 1)	0 (0, 1)	< 0.001	1 (0, 1)	< 0.001	0 (0, 3)	< 0.001
SOFA of renal	1 (0, 2)	1 (0, 2)	< 0.001	2 (1, 3)	< 0.001	1 (0, 2)	0.017
**Laboratory tests (Median (IQR))**							
Hb (g/dL)	9.9 (8.4, 11.5)	10.2 (8.4, 11.9)	< 0.001	10.4 (8.9, 12.1)	< 0.001	10.7 (9.1, 12.2)	< 0.001
MCH (pg)	30 (28.5, 31.3)	29.6 (28.1, 31)	< 0.001	29.8 (27.9, 31.5)	0.003	30.2 (29, 31.4)	< 0.001
WBC (10^9^/L)	14.3 (10.5, 19.2)	14.9 (10.3, 19.8)	0.085	13.9 (9.5, 19.3)	< 0.001	11.4 (7.3, 16.8)	< 0.001
PLT (10^9^/L)	163 (114, 225)	175 (123, 243)	< 0.001	179 (112, 256)	< 0.001	146 (88, 211)	< 0.001
NEU count (10^9^/L)	10.5 (7, 14.9)	10.6 (7, 15.5)	0.408	-	-	10 (6.2, 15.1)	0.017
LYM count (10^9^/L)	1 (0.6, 1.6)	0.9 (0.6, 1.4)	< 0.001	0.7 (0.4, 1.2)	< 0.001	0.6 (0.3, 0.9)	< 0.001
MON count (10^9^/L)	0.5 (0.3, 0.8)	0.6 (0.3, 1)	< 0.001	0.6 (0.4, 0.8)	< 0.001	0.4 (0.2, 0.7)	< 0.001
INR	1.3 (1.2, 1.6)	1.3 (1.2, 1.6)	0.45	1.7 (1.3, 2.6)	< 0.001	1.3 (1.2, 1.5)	< 0.001
PT (seconds)	14.7 (13, 17.7)	14.4 (12.9, 17.5)	0.11	19.2 (15.7, 27.5)	< 0.001	14.2 (12.7, 16.2)	< 0.001
PTT (seconds)	33.2 (28.4, 45.6)	33.5 (28.7, 56.5)	0.001	37.8 (31.9, 46.7)	< 0.001	32.9 (29.1, 38)	< 0.001
FIB (g/L)	2.3 (1.7, 3.4)	3.4 (2.1, 5.5)	< 0.001	4 (2.6, 5.6)	< 0.001	4.1 (3.1, 5)	< 0.001
D-Dimer (ng/mL)	3470.5 (1647, 6266)	2008 (971, 5230)	0.207	-	-	2161 (722, 5386)	0.135
Glucose (mmol/L)	8.3 (6.7, 11.0)	9.6 (7.6, 13.2)	< 0.001	7.4 (7, 9.2)	< 0.001	8.4 (6.6, 11.8)	0.959
BUN (mmol/L)	8.2 (5.7, 13.6)	9.3 (6.1, 16.1)	< 0.001	11.8 (7.3, 18.8)	< 0.001	10.4 (6.5, 16.9)	< 0.001
Creatinine(µmol/L)	106 (71, 159)	115 (80, 195)	< 0.001	141 (88, 248)	< 0.001	106 (69, 188)	0.626
TBIL (µmol/L)	12 (6.8, 25.7)	10.3 (6.8, 18.8)	< 0.001	13.7 (8.6, 29.1)	< 0.001	16.2 (10.4, 28.1)	< 0.001
DBIL (µmol/L)	30.8 (12, 68.4)	37.6 (15.4, 68.4)	0.287	10.3 (3.4, 27.4)	< 0.001	9.4 (5.4, 19)	< 0.001
ALT (U/L)	31 (18, 70)	31 (18, 63)	0.329	31 (19, 63)	0.858	33 (20, 58)	0.441
AST (U/L)	47 (27, 114)	50 (29, 100)	0.684	44 (25, 99)	< 0.001	43 (28, 85)	0.001
ALP (U/L)	82 (61, 120)	83 (61, 114)	0.742	102 (74, 152)	< 0.001	84 (60, 131)	0.384
TC (mmol/L)	3.7 (2.9, 4.4)	3.2 (2.4, 4.3)	0.079	2.7 (2.6, 2.8)	< 0.001	2.5 (1.9, 3.2)	< 0.001
TG (mmol/L)	1.4 (0.9, 2.3)	2.1 (1.3, 3.7)	< 0.001	1.4 (0.9, 2.1)	0.769	1.4 (0.9, 2.2)	0.901
pH	7.3 (7.2, 7.4)	7.3 (7.2, 7.3)	< 0.001	7.3 (7.3, 7.4)	< 0.001	7.4 (7.3, 7.4)	< 0.001
PaO_2_ (mmHg)	73 (45, 104)	53 (38, 80)	< 0.001	76 (61, 100)	< 0.001	103(81, 148)	< 0.001
Lactate (mmol/L)	2.3 (1.5, 3.7)	2.4 (1.6, 4.2)	0.015	2.4 (1.5, 3.8)	0.696	1.8 (1.2, 2.8)	< 0.001
PaO_2_/FiO_2_ ratio	160 (94, 254)	115 (74.3, 210)	< 0.001	170 (119, 312)	< 0.001	228 (158, 330)	< 0.001
Bicarbonate (mmol/L)	22 (20, 26)	21 (18, 24)	0.011	22 (19, 25)	0.149	20 (17, 23)	< 0.001
Potassium (mmol/L)	4.7 (4, 5.4)	4.3 (3.8, 5)	< 0.001	4.1 (3.7, 4.7)	< 0.001	3.8 (3.5, 4.2)	< 0.001
Sodium (mmol/L)	138 (135, 140)	137 (134, 141)	0.015	137 (134, 141)	< 0.001	138 (134, 142)	0.889
**Vital signs (Median (IQR))**							
Heart rate (bpm)	104 (90, 119)	104 (91, 119)	0.458	98 (85, 111)	< 0.001	94 (83, 104)	< 0.001
MAP (mmHg)	57 (51, 64)	60 (53, 65)	< 0.001	75 (69, 84)	< 0.001	86 (81, 91)	< 0.001
Temperature (℃)	37.4 (37.1, 38.1)	37.5 (37.1, 38.2)	< 0.001	36.9 (36.5, 37.4)	< 0.001	37.1 (36.7, 37.5)	< 0.001
Respiratory rate (bpm)	27 (24, 32)	28 (24, 33)	< 0.001	22 (19, 26)	< 0.001	19 (17, 22)	< 0.001
**Comorbidity diseases (n (%))**							
Hypertension	6894 (62.5)	791 (57.7)	< 0.001	196 (6.9)	< 0.001	736 (54.4)	< 0.001
Diabetes	3431 (31.1)	486 (35.4)	0.001	63 (2.2)	< 0.001	429 (31.7)	0.67
CKD	2444 (22.2)	325 (23.7)	0.205	436 (15.3)	< 0.001	119 (8.8)	< 0.001
Chronic liver disease	67 (0.6)	10 (0.7)	0.718	0 (0)	< 0.001	31 (2.3)	< 0.001
**Support treatment (n (%))**							
Mechanical ventilation	7297 (66.1)	1255 (91.5)	< 0.001	807 (28.3)	< 0.001	701 (51.8)	< 0.001
CRRT treatment	633 (5.7)	204 (14.9)	< 0.001	189 (6.6)	0.019	229 (16.9)	< 0.001
Heparin use	4844 (43.9)	772 (56.3)	< 0.001	287 (10.1)	< 0.001	1205 (89.1)	< 0.001
**Outcome (Median (IQR))**							
LOS of hospital (days)	10.6 (6.6, 17.7)	15.6 (8.7, 26.8)	< 0.001	9.3 (5.8, 15.6)	< 0.001	19.1 (11, 30.7)	< 0.001
LOS of ICU (days)	4.6 (2.9, 8.6)	7.2 (4, 14.5)	< 0.001	4.5 (2.9, 7.8)	0.066	10 (5.5, 18.1)	< 0.001
28-day mortality (%)	1961 (17.8)	332 (24.2)	< 0.001	605 (21.2)	< 0.001	334 (24.7)	< 0.001
ICU mortality (%)	3044 (27.6)	300 (21.9)	< 0.001	381 (13.4)	< 0.001	298 (22)	< 0.001
Hospital mortality (%)	2590 (23.5)	380 (27.7)	< 0.001	632 (22.2)	< 0.001	354 (26.2)	0.031

Note: * Compared with Derivation cohort group; - indicates no data for this item

Abbreviations: SIC: Sepsis-induced Coagulopathy; MIMIC-IV: Medical Information Mart for Intensive Care IV; eICU: eICU Collaborative Research Database; BMI: Body Mass Index; IQR: Inter Quartile Range; SOFA: Sequential Organ Failure Assessment; CNS: Central Nervous System; Hb: Hemoglobin; MCH: Mean Corpuscular Hemoglobin; WBC: White Blood Cell; PLT: Platelet; NEU：neutrophils; LYM: lymphocytes; MON: monocytes; INR: International Normalized Ratio; PT: Prothrombin Time; PTT: Partial Thromboplastin Time; FIB: Fibrinogen; BUN: Blood Urea Nitrogen; TBIL: total bilirubin; DBIL: direct bilirubin; ALT: Alanine aminotransferase; AST: Aspartate aminotransferase; ALP: Alkaline phosphatase; TC: Total cholesterol; TG: Triglycerides; pH: Potential of Hydrogen; PaO_2_: Partial pressure of oxygen; FiO_2_: Fraction of inspiration oxygen; MAP: Mean Arterial Pressure; CKD: Chronic kidney disease; CRRT: Continuous Renal Replacement Therapy; LOS: Length of hospital Stay; ICU: Intensive Care Unit

Among the derivation cohort, 6,240 (56.6%) patients were classified as having SIC, compared with 4,793 (43.4%) without SIC (NSIC). In the external validation cohorts, the cumulative incidence of SIC was 47.3% (649/1,371) in Cohort 1, 72.8% (2,074/2,848) in Cohort 2, and 57.6% (780/1,353) in Cohort 3. As summarized in Table S1, patients with SIC consistently differed from NSIC patients across all cohorts in most demographic and clinical parameters. Those with SIC generally exhibited more abnormal laboratory results, required organ support therapies more frequently, and had significantly higher 28-day mortality.

### Model performance comparison

Ultimately, seven key features were selected for model development: INR, lactate, platelet count, SOFA subscores (circulatory, renal, and respiratory), and mechanical ventilation status. Using these features, five ML models (LightGBM, RF, XGBoost, LR, and SVM) were developed to predict SIC occurrence within 7 days.

**Fig. 2:**
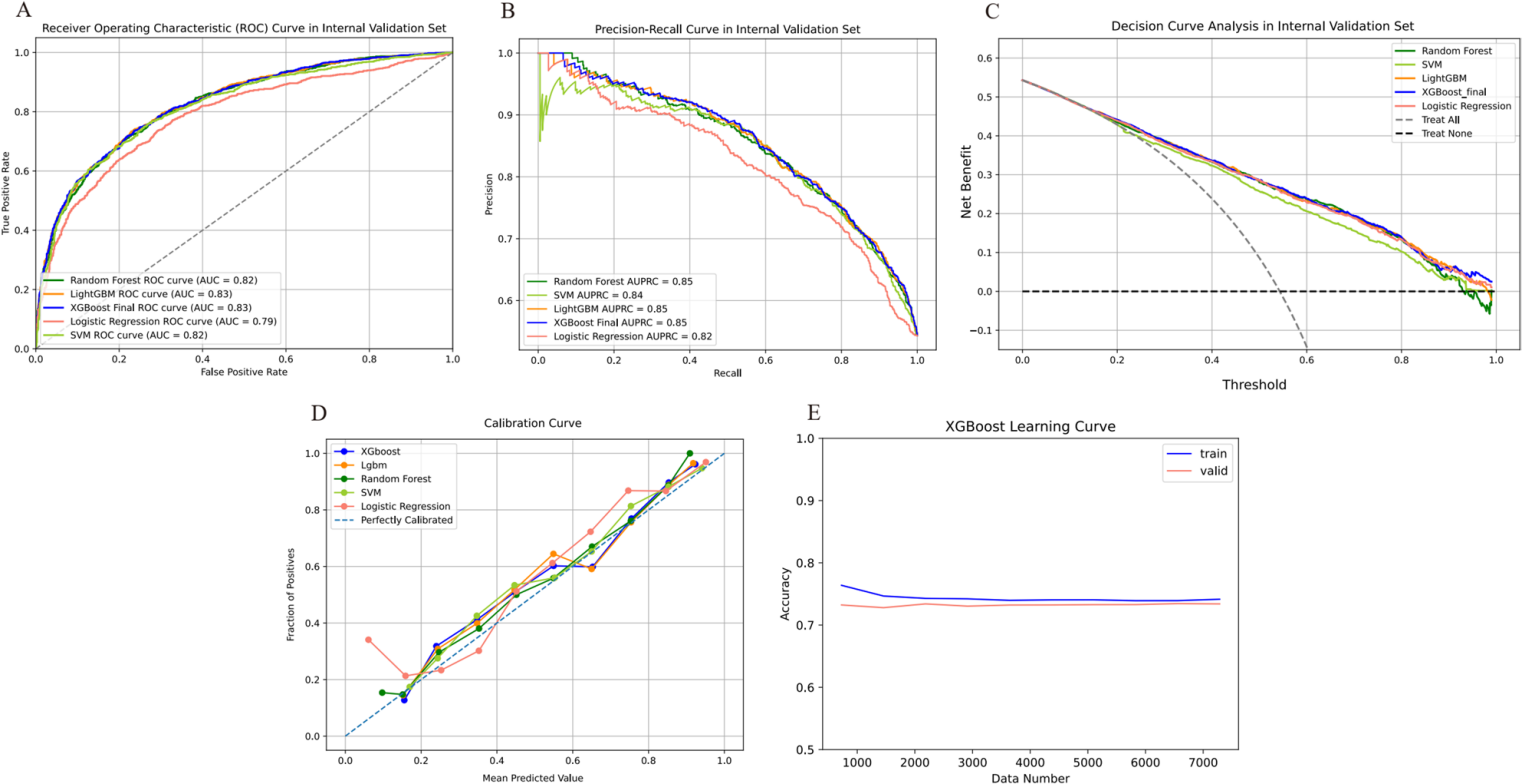
The performance and comparison of five different predictive models in internal validation set. (**A**) ROC curves for the internal validation set; (**B**) Precision-recall curves for the internal validation set; (**C**) Decision curve analysis for the internal validation set; (**D**) Calibration curve for the internal validation set; (**E**) XGBoost model’s learning curve on internal validation set. ROC: Receiver Operating Characteristic; AUC: Area Under Curve; XGBoost: Extreme Gradient Boosting; LightGBM: Light Gradient Boosting Machine; SVM: Support Vector Machine; AUPRC: Area Under the Precision-Recall Curve

On internal validation set, the XGBoost model demonstrated the best performance. Model performance was evaluated using AUROC curves, precision-recall (PR) curves, calibration curves, and decision curve analysis (DCA). And the quantitative metrics of the calibration curves for each cohort model are shown in Table S4. The XGBoost model achieved an AUC of 0.83 (95%CI 0.811–0.844), outperforming the other models. The AUC values for the remaining models were as follows: LightGBM, 0.83 (95%CI 0.809–0.844); RF, 0.82 (95%CI 0.806–0.840); SVM, 0.82 (95%CI 0.799–0.833); and LR, 0.79 (95%CI 0.766–0.804) (Fig. 2A-C). The discriminatory performance of all five models is summarized in Table 2 and Table S5.

**Table 2:** Model performance for different machine learning models in derivation and external validation datasets for early-stage SIC onset prediction

Dataset	Machine Learning Models	Accuracy	F1 Score	AUC	AUPRC	Sensitivity	Specificity	PPV	NPV
**Internal validation**	**XGBoost**	0.745	0.754	0.827	0.854	0.719	0.776	0.793	0.699
	**RF**	0.742	0.752	0.823	0.852	0.719	0.769	0.787	0.697
	**LightGBM**	0.746	0.756	0.827	0.853	0.723	0.774	0.791	0.702
	**LR**	0.712	0.715	0.785	0.821	0.668	0.764	0.771	0.659
	**SVM**	0.737	0.743	0.817	0.840	0.700	0.780	0.791	0.686
**Internal test**	**XGBoost**	0.740	0.740	0.820	0.845	0.705	0.778	0.780	0.703
	**RF**	0.734	0.738	0.810	0.839	0.711	0.760	0.767	0.703
	**LightGBM**	0.736	0.738	0.821	0.845	0.705	0.771	0.774	0.701
	**LR**	0.726	0.723	0.789	0.809	0.677	0.782	0.776	0.685
	**SVM**	0.740	0.739	0.818	0.844	0.697	0.789	0.786	0.700
**External validation** **(MIMIC-IV 3.1)**	**XGBoost**	0.853	0.872	0.927	0.954	0.856	0.850	0.889	0.808
	**RF**	0.829	0.844	0.909	0.941	0.792	0.880	0.903	0.751
	**LightGBM**	0.830	0.843	0.917	0.947	0.784	0.894	0.912	0.747
	**LR**	0.819	0.829	0.912	0.941	0.751	0.915	0.926	0.724
	**SVM**	0.772	0.796	0.843	0.886	0.762	0.785	0.833	0.702
**External validation** **(eICU)**	**XGBoost**	0.809	0.871	0.859	0.942	0.885	0.600	0.859	0.654
	**RF**	0.787	0.850	0.846	0.936	0.826	0.678	0.876	0.587
	**LightGBM**	0.783	0.846	0.852	0.939	0.810	0.711	0.885	0.576
	**LR**	0.715	0.779	0.810	0.905	0.683	0.801	0.904	0.480
	**SVM**	0.689	0.745	0.841	0.928	0.619	0.879	0.934	0.457
**External validation (ZhongDa Hospital)**	**XGBoost**	0.630	0.571	0.825	0.590	0.931	0.522	0.412	0.955
	**RF**	0.629	0.569	0.819	0.592	0.925	0.523	0.411	0.951
	**LightGBM**	0.673	0.594	0.824	0.586	0.905	0.590	0.442	0.945
	**LR**	0.663	0.583	0.818	0.586	0.890	0.582	0.433	0.936
	**SVM**	0.702	0.600	0.803	0.535	0.845	0.651	0.465	0.922

**Abbreviations**: SIC: Sepsis induced coagulopathy; AUC: Area Under Curve; AUPRC: Area Under the Precision-Recall Curve; PPV: Positive Predictive Value; NPV: Negative Predictive Value; XGBoost: Extreme Gradient Boosting; RF: Random Forest; LightGBM: Light Gradient Boosting Machine; LR: Logistics Regression; SVM: Support Vector Machine; MIMIC-IV: Medical Information Mart for Intensive Care IV; eICU: eICU Collaborative Research Database

The calibration performance and goodness-of-fit of the XGBoost model were systematically assessed. On internal validation, the calibration curve aligned closely with the ideal diagonal across most risk intervals, indicating clinically reliable predicted probabilities and well-calibrated performance (Fig. 2D). The learning curve showed robust convergence and satisfactory fit, reflecting strong generalizability and stability (Fig. 2E). Given these findings, the XGBoost model was selected as base model for further hyperparameter tuning via Bayesian optimization on the internal test set. The optimized XGBoost model exhibited superior performance on the internal test set, achieving an AUC of 0.82 (95% CI 0.795–0.844), an AUPRC of 0.845 (95% CI 0.816–0.870), accuracy of 74%, sensitivity of 70.5%, specificity of 77.8%, PPV of 0.78, NPV of 0.703, and an F1-score of 0.74 (Table 2, Table S6, and Figure S5).

### Model explanation

**Fig. 3:**
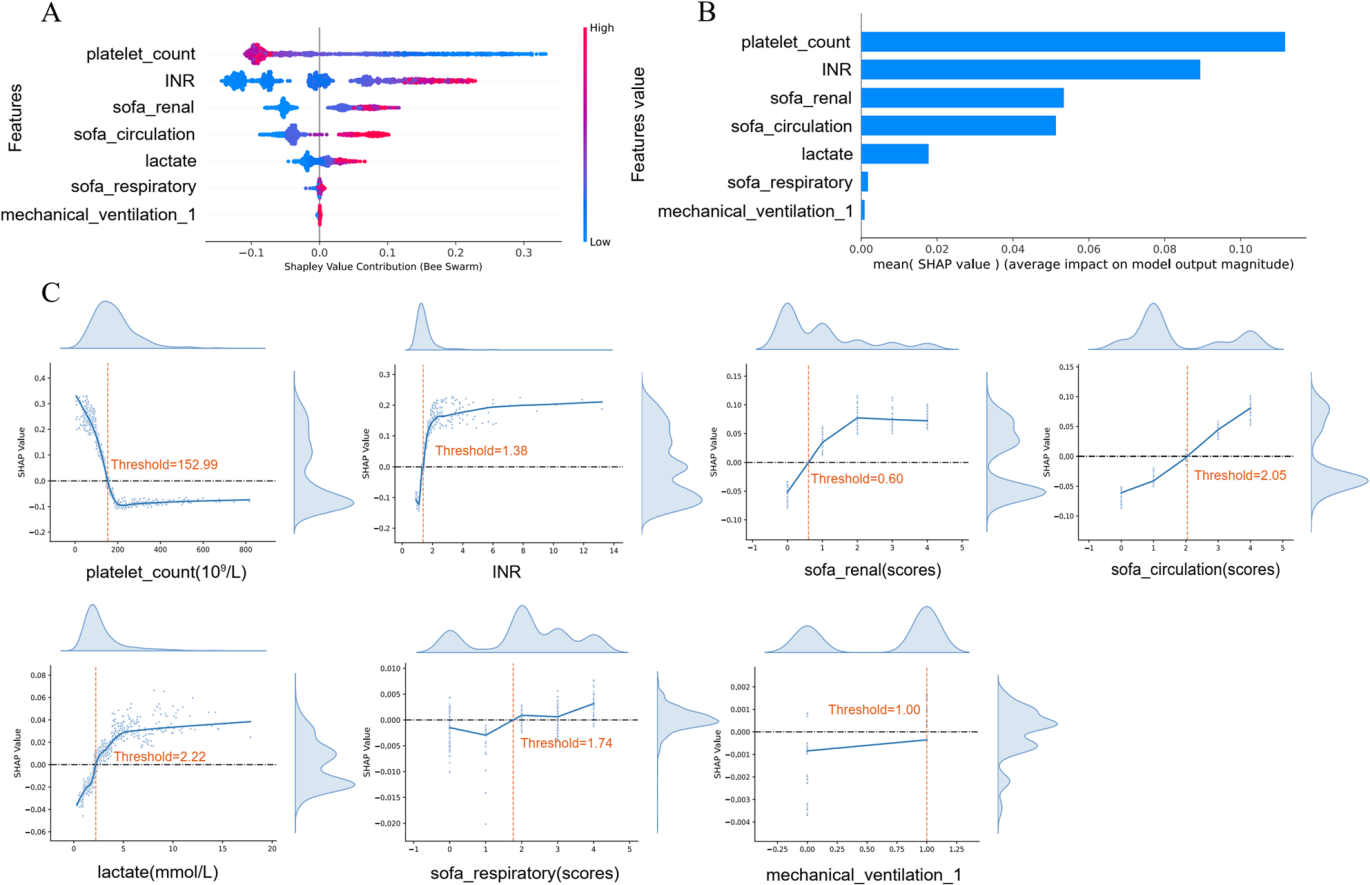
Global model interpretation using the SHAP method in internal test set. (**A**) SHAP summary bar plot; (**B**) SHAP summary dot plot; A dot is made for SHAP value in the model for each single patient, so each patient has one dot on the line for each feature. The colors of the dots demonstrate the actual values of the features for each patient, as red means a higher feature value and blue means a lower feature value. The dots are stacked vertically to show density. (**C**) SHAP dependence plot. Each dependence plot shows how a single feature affects the output of the prediction model, and each dot represents a single patient. Note: These attributions reflect model behavior and do not necessarily imply biological causality. INR: International Normalized Ratio; sofa: Sequential Organ Failure Assessment; SHAP: SHapley Additive explanation

To provide post-hoc model-centric attribution, the SHAP method was applied to characterize the final model’s internal decision patterns. It is important to note that SHAP identifies feature contributions to the model’s output rather than providing actionable causal explanations. Global feature attribution was used to describe the model’s overall behavior across the training set. As depicted in the SHAP summary plots, features were ranked by their mean absolute SHAP values, reflecting their relative contribution to the model’s predictions (Fig. 3A-B). SHAP dependence plots (Fig. 3C) were utilized to illustrate the numerical relationship between the seven clinical features and their corresponding attribution scores. For instance, in this specific dataset and model architecture, adult patients with PLT ≤ 153 × 10⁹/L or lactate ≥ 2.22 mmol/L exhibited positive SHAP values, which influenced the model toward an ‘SIC’ classification.

**Fig. 4:**
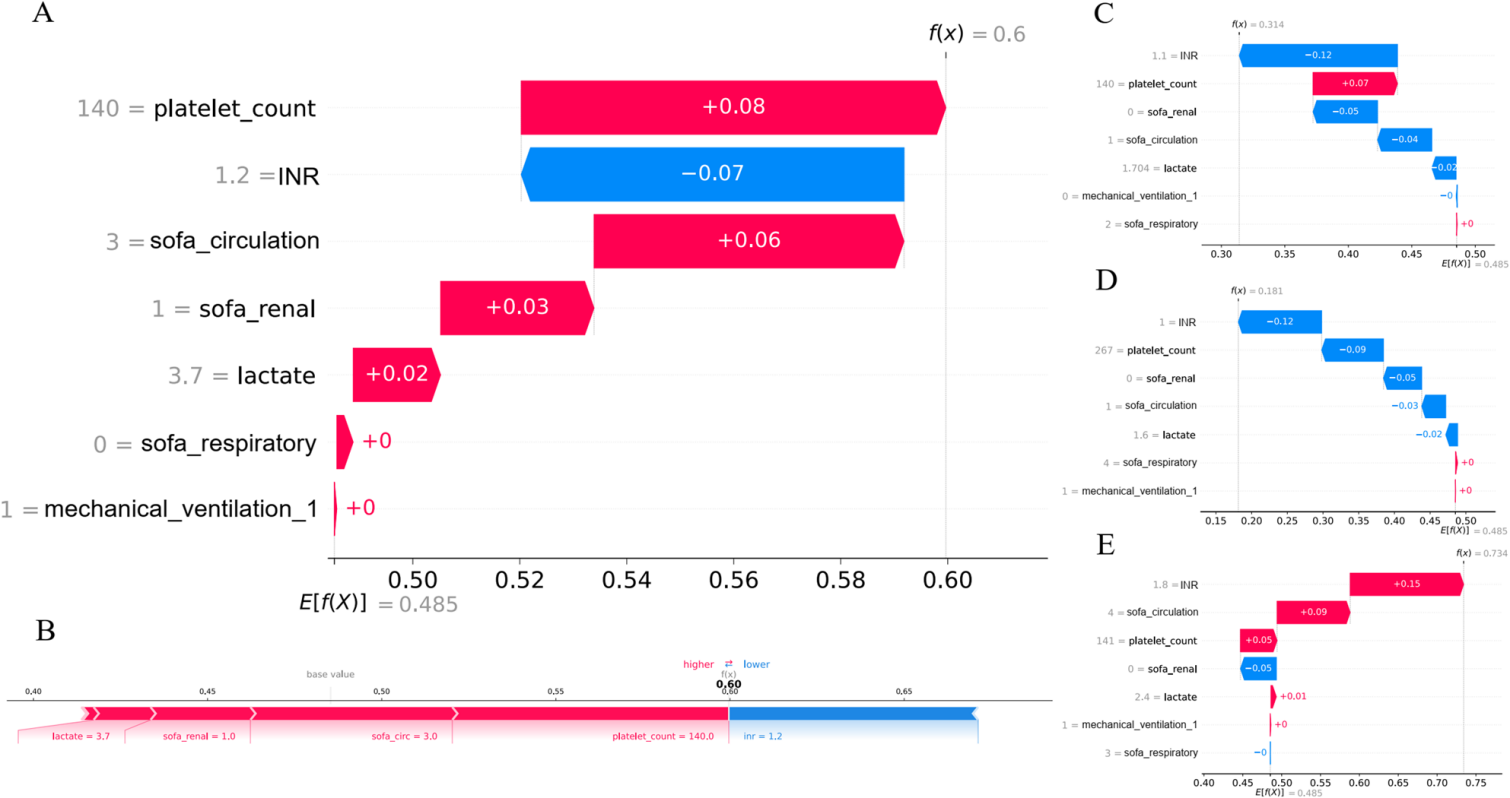
Local model explanation by the SHAP method. Waterfall plot and evolution of risks contributed by each feature for a specific patient (**A**), and the Force plot corresponding to this patient (**B**). Interpretability analysis of waterfall plots for 3 independent samples at low or high risk of developing SIC (**C**-**E**). Each patient is represented by the x-axis, while the contribution of features is represented by the y-axis: the larger the red part of each patient, the more likely it is to be judged as “SIC”. Note: These attributions reflect model behavior and do not necessarily imply biological causality. INR: International Normalized Ratio; sofa: Sequential Organ Failure Assessment; SIC: Sepsis-Induced-Coagulopathy; SHAP: SHapley Additive explanation

Individual-case attribution was performed to demonstrate how the model integrates specific features for a single prediction. In these visualizations, features highlighted in red push the risk score higher, whereas those in blue lower it. Figures 4A-B present the waterfall and force plots for an exploratory case with a predicted SIC probability of 60%, reflecting the model’s internal logic for that specific data point. Figures 4C-E present additional waterfall plots for representative cases.

### External validation of the final model

**Fig. 5:**
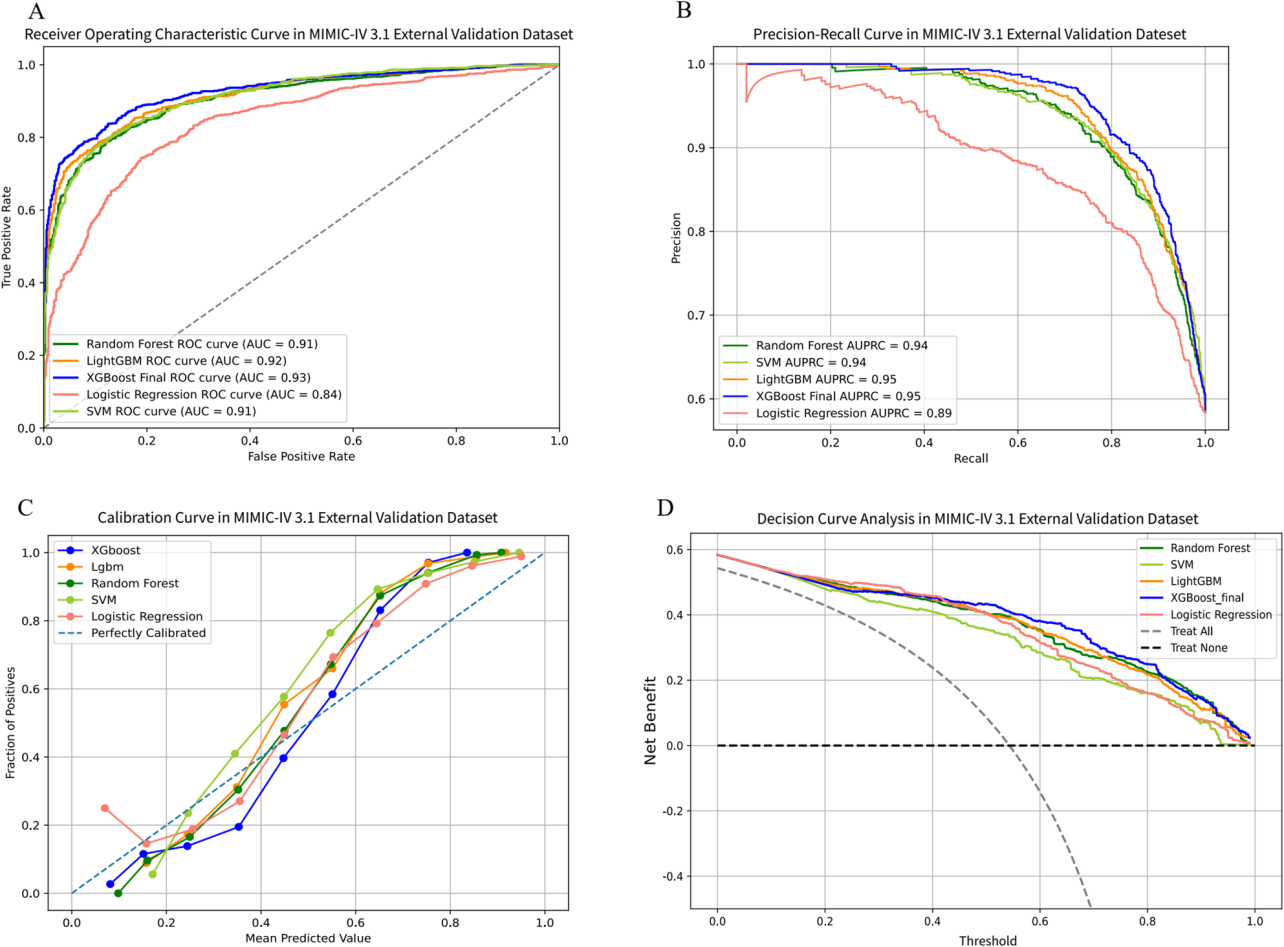
The performance and comparison of five different predictive models in MIMIC-IV 3.1 external validation dataset. (**A**) ROC curves for the MIMIC-IV 3.1 external validation set; (**B**) Precision-recall curves for the MIMIC-IV 3.1 external validation set; (**C**) Decision curve analysis for the MIMIC-IV 3.1 external validation set. ROC: Receiver Operating Characteristic; AUC: Area Under Curve; XGBoost: Extreme Gradient Boosting; LightGBM: Light Gradient Boosting Machine; SVM: Support Vector Machine; AUPRC: Area Under the Precision-Recall Curve. MIMIC-IV: Medical Information Mart for Intensive Care IV

**Fig. 6:**
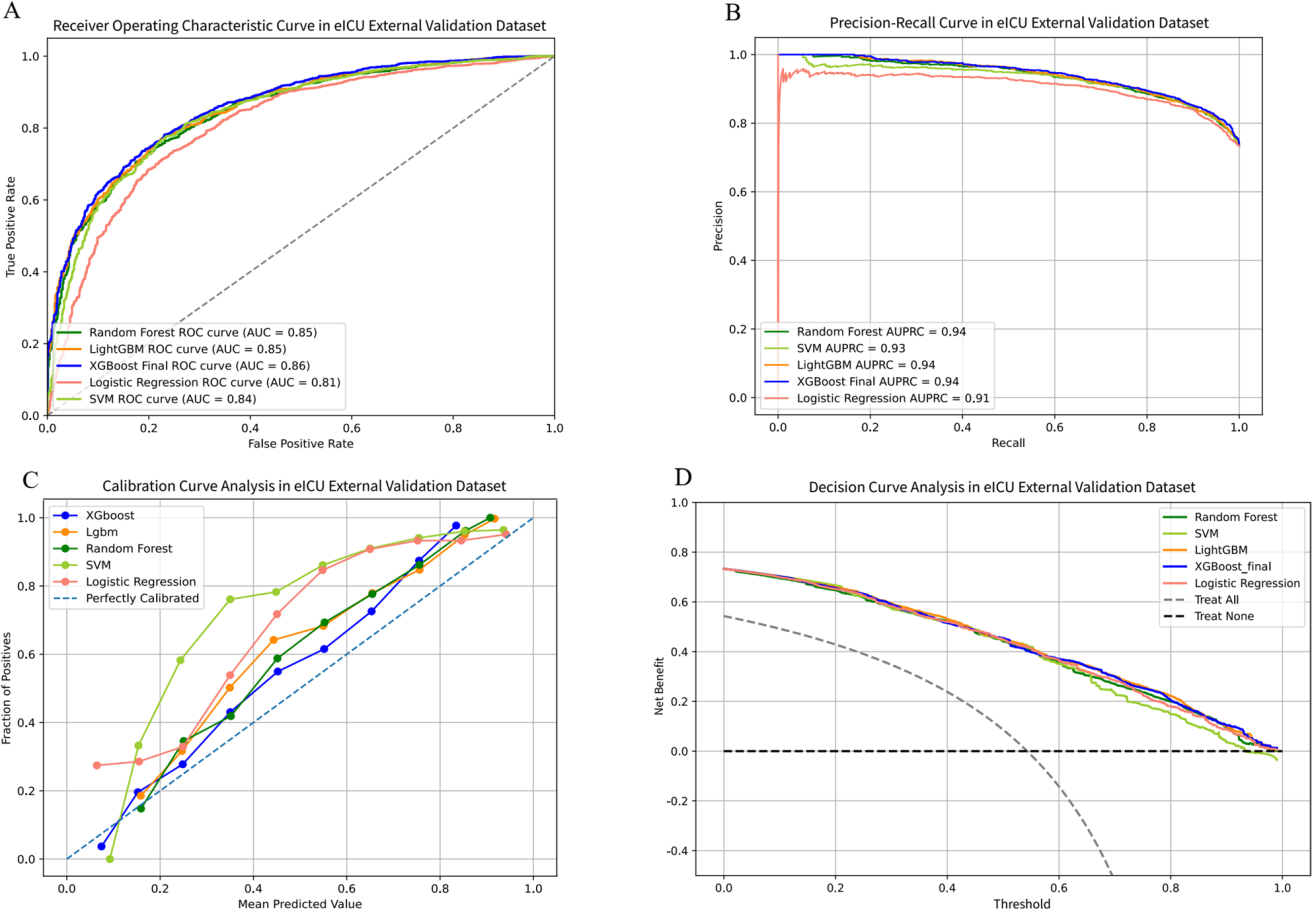
The performance and comparison of five different predictive models in eICU external validation dataset.(**A**) ROC curves for the eICU external validation set; (**B**) Precision-recall curves for the eICU external validation set; (**C**) Decision curve analysis for the eICU external validation set. ROC: Receiver Operating Characteristic; AUC: Area Under Curve; XGBoost: Extreme Gradient Boosting; LightGBM: Light Gradient Boosting Machine; SVM: Support Vector Machine; AUPRC: Area Under the Precision-Recall Curve; eICU: eICU Collaborative Research Database

**Fig. 7:**
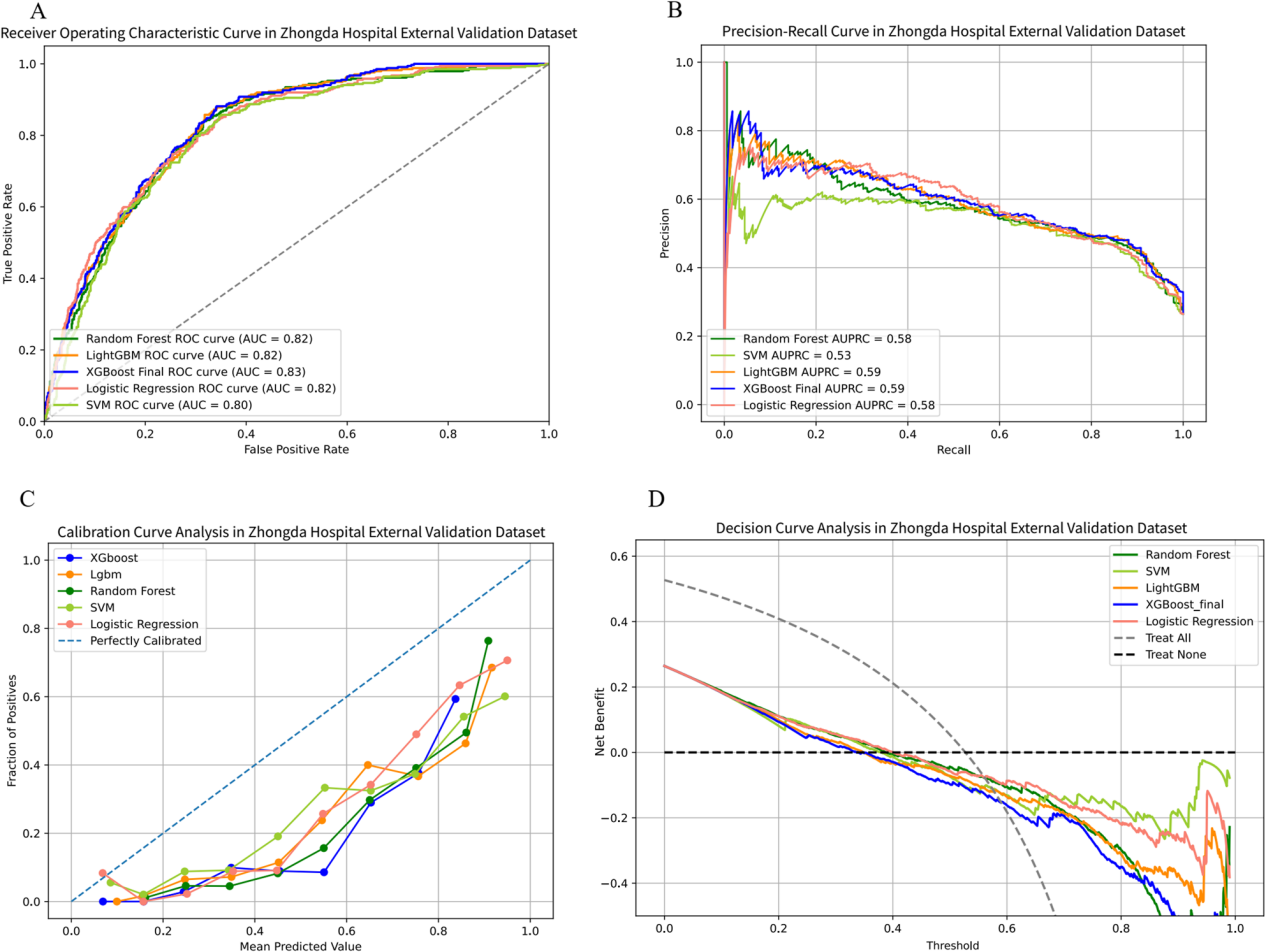
The performance and comparison of five different predictive models in Zhongda hospital external validation set. (**A**) ROC curves for the Zhongda hospital external validation set; (**B**) Precision-recall curves for the Zhongda hospital external validation set; (**C**) Decision curve analysis for the Zhongda hospital external validation set. ROC: Receiver Operating Characteristic; AUC: Area Under Curve; XGBoost: Extreme Gradient Boosting; LightGBM: Light Gradient Boosting Machine; SVM: Support Vector Machine; AUPRC: Area Under the Precision-Recall Curve

The performance of the final model was evaluated on three distinct external validation cohorts. In External Validation Cohort 1, the XGBoost model achieved an AUC of 0.93 (95% CI 0.916–0.939), an AUPRC of 0.95 (95% CI 0.946–0.962), an accuracy of 85.3%, a sensitivity of 85.6%, a specificity of 85%, a PPV of 0.889, an NPV of 0.808, and an F1-score of 0.872 (Table 2, Table S7; Fig. 5). In External Validation Cohort 2, the model attained an AUC of 0.859 (95% CI 0.845–0.873), an AUPRC of 0.942 (95% CI 0.933–0.949), an accuracy of 80.9%, a sensitivity of 88.5%, a specificity of 60%, a PPV of 0.859, an NPV of 0.654, and an F1-score of 0.871 (Table 2, Table S7; Fig. 6). In External Validation Cohort 3, the model yielded an AUC of 0.83 (95% CI 0.801–0.846), an AUPRC of 0.59 (95% CI 0.535–0.645), an accuracy of 63.0%, a sensitivity of 93.1%, a specificity of 52.2%, a PPV of 0.412, an NPV of 0.955, and an F1-score of 0.571 (Table 2, Table S7; Fig. 7).

Across all three external validation cohorts, the XGBoost model consistently outperformed other models. Although certain metrics including AUPRC, accuracy, specificity, PPV, and F1-score were lower in External Validation Cohort 3 relative to the internal test set, the model demonstrated comparable or superior performance in the other external cohorts. Collectively, these results indicate that the final model generalizes effectively and retains robust discriminative ability across diverse external datasets. We further compared the predictive performance of the final XGBoost model against individual baseline clinical parameters including circulatory, renal, and respiratory SOFA scores, platelet count, INR, lactate level, and the day 1 SIC score for predicting early SIC occurrence, and quantified the incremental value of the model. The XGBoost model consistently surpassed all individual predictors on both the internal test set and the external validation cohorts (Table S8, Figure S6, Figure S7). Since SIC can develop at any point following ICU admission in septic patients, we further explored the model’s performance in predicting late-onset SIC (occurring between Day 4 and Day 7). Detailed results are provided in Table S9. The model achieved an AUC of 0.503 (95% CI 0.475–0.532) on the internal test set, and 0.475 (95% CI 0.440–0.507), 0.53 (95% CI 0.481–0.578), and 0.598 (95% CI 0.515–0.676) across External Validation Cohorts 1, 2, and 3, respectively. These results indicate that the XGBoost model lacks discriminative power for predicting late-onset SIC.

### Model robustness and sensitivity analyses

Multi-dimensional sensitivity analyses confirmed the high robustness of the Early-SIC model across diverse and complex clinical scenarios:

Assessment of Baseline Comorbidities: Subgroup analyses of patients with chronic medical histories revealed the model’s ability to adjust for varying physiological baselines. Mean Risk Difference (MRD) analysis showed significant negative risk offsets in the CKD subgroup (*P* < 0.001 in both MIMIC-IV and eICU cohorts), with consistent risk-adjustment trends also observed in Hypertension (*P* = 0.0026 in eICU) and Diabetes subgroups (Table S10, Figure S8). This indicates that the model internalized baseline aberrations caused by chronic organ dysfunction, effectively distinguishing chronic offsets from acute sepsis-induced insults. Despite these baseline confounders, discriminative performance remained stable, with no statistically significant differences in AUC between the full cohort and subgroups excluding CKD, hypertension, or diabetes (all *P* > 0.05) (Table S11).

Control of Data Missingness Bias: Evaluation of missing bilirubin data (38.7%) showed no systematic predictive bias in most cohorts, as risk score distributions between missing and non-missing groups were not significantly different (*P* > 0.05) (Table S10, Figure S8). Further comparison between the full cohort and the complete-case cohort (non-missing data) revealed negligible fluctuations in AUC (ΔAUC ranging from − 0.0084 to 0.057, all *P* > 0.5), confirming that the conservative clinical assumption of assigning a sub-score of 0 for missing values did not introduce significant bias (Table S8).

Validation of Independence from Clinical Interventions: The model exhibited highly consistent performance across subgroups stratified by Heparin use, MV, and CRRT treatment (Table S12). Multicenter MRD analysis showed that, with few exceptions, the average risk difference between exposed and non-exposed groups remained statistically insignificant (*P* > 0.05) (Table S10, Figure S8). Additionally, AUC comparisons between the full cohort and non-exposed subgroups (e.g., Non-Heparin) showed no significant variations (*P* > 0.8 across centers), indicating that the predictive signals are primarily driven by intrinsic biological markers rather than clinician intervention patterns (Table S11).

Lead-time Analysis: To evaluate prospective early-warning utility, patients meeting SIC criteria within 24 h of admission were excluded. This conservative approach led to high exclusion rates (72.82% in eICU and 72.08% in MIMIC-IV 3.1), indicating that most SIC cases had manifested upon admission, thereby weakening the predictive signal in remaining subsets. Consequently, AUCs followed a signal-dependent gradient: remaining robust in the derivation (0.864) and Zhongda cohorts (0.706), while falling to 0.562 and 0.517 in the high-exclusion eICU and MIMIC-IV 3.1 datasets. In the Zhongda cohort, despite a high specificity (0.878), the PPV was 0.151 due to a low positive rate (7.30%). Despite these challenges in signal-sparse environments, the model’s performance in key cohorts confirms its prospective predictive value (Table S13).

### Potential research applications and hypothesis-generating framework

To explore the predictive implications of the developed framework, we evaluated its performance using a combined dataset of 16,605 septic patients across all cohorts. Supplementary Table S14 summarizes the optimal prediction thresholds, Youden indices, and standard performance metrics (AUC, sensitivity, specificity, accuracy, PPV, and NPV). For the purpose of this exploratory analysis, a threshold probability of 0.54 was utilized to identify potential high-risk SIC profiles within the study population.

**Fig. 8:**
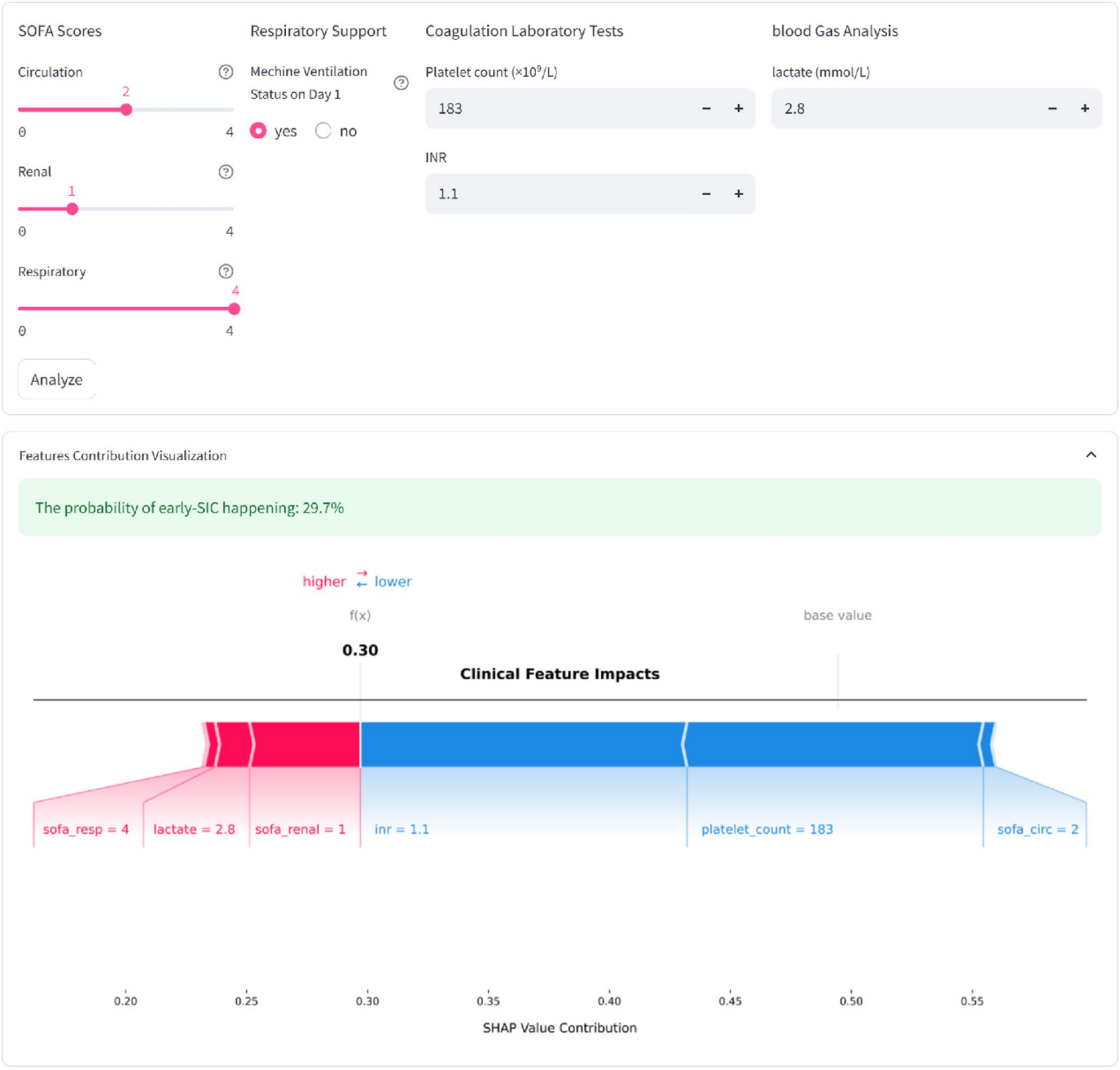
Application of a web-based predictor on the risk of sepsis induced coagulopathy in sepsis patients. The final XGBoost model developed in this study is based on seven features that can effectively predict the risk of SIC in sepsis patients. After inputting the actual values of these seven features, the application automatically calculates and displays the probability that the patient will develop SIC. Meanwhile, the force diagram for a single sepsis patient shows the features that help determine “SIC”: the red features on the left are those that push the prediction into the “SIC” category, while the blue features on the right are those that push the prediction into the “non-SIC” category. XGBoost, extreme gradient boosting. The website that predicts the risk of SIC in sepsis patients is https://early-sic-prediction-webpage.streamlit.app/

As a proof-of-concept for this hypothesis-generating framework, we have developed an interactive web-based demonstration (Fig. 8). This interface serves as a prototype to illustrate how the seven clinical features could be integrated to estimate the risk of SIC development within a 72-hour window. To enhance interpretability, the application generates a SHAP force plot for individual cases, providing a visual representation of how specific features might influence risk scores. This exploratory tool is intended for research purposes and is accessible at: https://early-sic-prediction-webpage.streamlit.app/.

Building upon this framework, we propose a proposed stratified intervention pathway driven by high-risk alerts. To balance clinical utility with the operational realities of the ICU, the model is designed to perform a single risk assessment specifically at the critical 24-hour mark following admission. This strategic timing aims to provide timely insights while mitigating the risk of alarm fatigue among healthcare providers. In this workflow, a “high-risk” prediction serves as a clinical trigger for evidence-based diagnostic escalation and preemptive physiological optimization. By defining tiered interventions tailored to specific risk stratifications, this pathway provides a structured roadmap for future research to evaluate whether such proactive decision-support mechanisms can effectively translate statistical accuracy into tangible clinical benefits (Figure S9).

## Discussion

This study demonstrates the feasibility of using a machine learning framework to identify the risk of early-onset SIC in septic patients. By incorporating seven readily available clinical features, the model exhibited robust performance across internal and external validation cohorts. Given its parsimonious structure and reliance on routine variables, this approach provides a potential foundation for future clinical decision-support research.

In this study, the cumulative incidence of SIC was 56.6% in the derivation cohort and 62.9% in the external validation cohort, consistent with previous reports [42–44]. Significant differences were observed between SIC and non-SIC groups in laboratory parameters, organ function, and support therapy. Notably, SIC patients had significantly worse outcomes, including heightened 28-day mortality, reaffirming SIC as an independent risk factor for adverse prognosis [13]. Therefore, early identification of high-risk patients is essential for timely intervention and improved outcomes [26].

Current clinical practice lacks effective tools for early SIC prediction in septic patients. While traditional scores like the SIC score offer high sensitivity, their suboptimal specificity limits precise early risk stratification [14]. For instance, the SIC triad may miss patients with early hyperfibrinolysis or elevated D-dimer but normal platelet counts, leading to false negatives [45–47]. INR specificity is reduced by confounders such as liver dysfunction, vitamin K administration, warfarin use, or transfusion [48, 49]. The SOFA score can disproportionately influence the overall score, potentially classifying severe organ dysfunction as coagulopathy even when actual coagulation parameters are only mildly abnormal [50]. Furthermore, anticoagulation or platelet transfusion prior to testing can mask coagulopathy progression, impairing the accurate prediction of imminent SIC [51, 52].

With the rapid advancement of artificial intelligence, ML has become a key technology for developing clinically reliable and accurate predictive models [53]. ML algorithms reduce subjective intervention and excel at handling complex, high-dimensional clinical data, achieving superior predictive performance compared to traditional models [54]. Developing a useful model requires balancing predictive accuracy with clinical utility, which hinges on methodological framework, feature selection, and validation performance.

Among five representative ML models compared, XGBoost performed best, achieving AUCs of 0.83 (internal validation) and 0.82 (internal test), and an AUPRC of 0.85 on both sets. It also maintained a good clinical balance, with sensitivity (0.70,0.72), specificity (0.77,0.78), PPV (0.78,0.79), and NPV (0.70) all within applicable ranges, indicating its suitability for clinical decision-making.

XGBoost, a powerful ensemble learning algorithm based on Gradient Boosted Decision Trees (GBDT), offers enhanced precision, speed, and robustness over traditional gradient boosting [55]. In this study, the model demonstrated strong clinical reliability, with calibration curves closely following the ideal line across most risk intervals. Furthermore, learning curves indicated robust performance and low overfitting risk, as the accuracy of training and validation sets converged and stabilized with increasing sample size, reflecting excellent generalization capability and model stability. The XGBoost model demonstrated strong predictive performance for early-onset SIC across external validation cohorts, achieving high AUC values of 0.93 in External Validation Cohort 1 and 0.86 in Cohort 2, along with robust results in AUPRC, accuracy, sensitivity, specificity, PPV, and NPV.

In the Zhongda Hospital validation cohort, while the AUC (0.83), sensitivity (0.931), and NPV (0.955) remained acceptable, we observed a notable decline in AUPRC and PPV. Beyond the pronounced class imbalance (shifting from a near 1:1 ratio on Day 1 to 1:3 by Day 3), this attenuation may be primarily attributed to the high prevalence of early heparin administration. Specifically, heparin usage was 43.9% in the derivation cohort compared to over 88% in the Zhongda Hospital cohort. To further investigate the impact of heparin on model performance, we conducted supplemental sensitivity analyses. The results demonstrated highly consistent discriminative performance between the heparin group (AUC 0.8543) and the non-heparin group (AUC 0.8237) within the Zhongda Hospital cohort. Furthermore, no statistically significant difference in AUC was found between the full cohort and the non-heparin subgroup (*P* = 0.8549). These findings suggest that the Early-SIC model captures endogenous, early-stage pathophysiological signals that manifest prior to clinical intervention, rendering the model’s ranking capability (AUC) robust to therapeutic confounding. However, acting as a “pathophysiological modifier”, heparin may improve sepsis outcomes and effectively prevent or reverse the progression of SIC in high-risk individuals [56–58]. This timely intervention successfully converts “potential true positives” (identified by the model) into “actual negatives,” thereby diluting the observed positive prevalence at the output stage. This is particularly evident in the lead-time analysis, where the positive rate fell to 7.30%, causing a mathematical drop in precision-based metrics (PPV and AUPRC). Consequently, the fluctuations in specific metrics observed in patient cohorts receiving early heparin intervention do not represent a limitation in generalizability or a flaw in predictive logic. Instead, they reflect the dynamic interplay between clinical early-warning and therapeutic intervention. When the model accurately identifies high-risk candidates, prompt heparin administration may halt the progression toward overt SIC, statistically diluting the positive outcome distribution. This observation reinforces the primary objective of this study: to facilitate early identification and prompt intervention to preemptively alter disease trajectories. These findings highlight the necessity of accounting for the “treatment effect” and its dynamic modification of outcome distributions when evaluating clinical early-warning tools.

The accurate definition of baseline SOFA scores is the cornerstone of ensuring the validity of the sepsis study population and is central to the diagnostic efficacy of SIC. To minimize the interference of chronic underlying diseases on the assessment of acute organ dysfunction, we implemented a rigorous baseline SOFA scoring strategy. This involved excluding patients who presented with severe end-stage organ failure (e.g., decompensated cirrhosis, active malignancies) or acute non-infectious insults (e.g., major trauma, hemorrhagic shock) at the time of ICU admission. Furthermore, sensitivity analyses demonstrated that the model’s predictive performance remained highly stable even after excluding patients with common chronic comorbidities such as CKD, hypertension, and diabetes. This confirms that the early-warning signals captured by the Early-SIC model primarily reflect acute pathophysiological changes induced by infection, rather than the patients’ chronic baseline states.

Regarding the common challenge of missing total bilirubin data in electronic health records (which accounted for 38.7% in our derivation cohort), we adopted a clinically recognized conservative imputation strategy: missing values were interpreted as the absence of known severe hepatic dysfunction on that calendar day, and the liver sub-score was consequently assigned a value of 0 [35, 36]. This strategy ensures the continuity of scoring and maximizes the cohort size, aligned with the clinical rationale that physicians are unlikely to order bilirubin tests for patients lacking signs of jaundice or hepatic impairment. To address potential concerns regarding imputation bias, we constructed a “complete-case” sub-cohort consisting only of patients with no missing bilirubin measurements throughout the observation period. Re-validating the model on this sub-cohort revealed no significant difference in predictive accuracy, providing strong evidence that the missing data did not introduce systematic bias.

Several studies have developed predictive models for SIC: Tan et al. developed a RF-based ML model using eight features to predict SIC risk, identifying APTT as the most important predictor, followed by lactate and urea nitrogen [25]. Yutaka et al. built a LightGBM-based model for early overt DIC warning, incorporating 21 features [26]. They reported the circulation SOFA score, lactate, and 24-hour urine output as the top predictors, with additional contributions from creatinine, respiratory rate, and total SOFA score. Zhao et al. utilized the CatBoost algorithm with time-series data for dynamic SIC prediction [24]. Their model highlighted coagulation markers (PLT, INR, and PT) as the most influential, followed by renal function indicators (urine output and creatinine), along with other variables including the circulation SOFA score and lactate. Cui et al. proposed a novel ODE-RNN model to predict SIC and sepsis-associated DIC 8n (1 ≤ *n* ≤ 6) hours before onset, identifying PLT, D-dimer, INR, plateletcrit (PCT), fibrinogen (FIB), and fibrin degradation products (FDP) as the most valuable predictors for DIC [23]. Li et al. identified shock, PLT, and INR as independent predictors of SIC and constructed a nomogram for estimating individual probabilities of SIC occurrence [44].

Our SIC prediction model utilized seven features: PLT, INR, renal SOFA score, circulation SOFA score, lactate, respiratory SOFA score, and MV status. Feature importance analysis ranked PLT and INR as the most influential predictors, followed by renal SOFA (renal function) and circulation SOFA (hemodynamic status). Respiratory SOFA and mechanical ventilation status had comparatively lower impact. This feature set al.igns with those identified in previous studies [23–26, 44], comprising routine clinical parameters—coagulation markers, organ dysfunction scores, lactate—that are biologically linked to SIC pathogenesis. This consistency reinforces the biological plausibility and representativeness of our model.

An ideal predictive model should capture the patient’s intrinsic pathophysiological risk rather than merely reflecting clinical management patterns. To isolate the model’s reliance on biological signals and mitigate potential confounding from therapeutic interventions, we conducted a sensitivity analysis by excluding intervention-related features mechanical ventilation from the feature set. Following this exclusion, the “physiology-only” model maintained a stable AUC. This confirms that the core predictive power of the Early-SIC model is derived from fundamental biological markers—such as platelets, INR, lactate, and creatinine—rather than learned patterns of clinician intervention. Such independence ensures the model’s robustness and generalizability across diverse clinical settings.

Our study demonstrates that while the model exhibits robust predictive performance for early-onset SIC (≤ 72 h), it lacks discriminative power for late-onset SIC (> 72 h). This discrepancy likely reflects fundamental differences in the underlying pathophysiological mechanisms between these two SIC subtypes. Early-onset SIC is likely driven more directly by the initial inflammatory surge and endothelial injury characteristic of early sepsis; it progresses rapidly, with risk factors often already manifest upon ICU admission. In contrast, the development of late-onset SIC may be more heavily influenced by subsequent clinical events, such as secondary infections, emerging organ dysfunctions, persistent nutritional status, and complex hospital-based interventions (e.g., blood transfusions or surgery). These evolving factors are difficult to capture fully using only data from the initial 24 h of ICU admission. This finding aligns with clinical intuition that predicting delayed complications is inherently more challenging. Consequently, we have explicitly defined this model as an early risk-warning tool designed to support decision-making during the “golden window” of intervention.

The transition of a predictive model to clinical practice requires defining an optimal cutoff. In this study, candidate thresholds identified by the Youden index showed variation from 0.51 to 0.63 across datasets, with observed variation attributable to population heterogeneity. However, the corresponding Youden indices demonstrated strong consistency, clustering between 0.50 and 0.55 in five of the six cohorts, which confirms the model’s stable discriminative performance and generalizability. The threshold of 0.54 was selected for several reasoned considerations. Statistically, it resides within the constrained range (0.51–0.56) consistent with our core validation sets. Clinically, it achieves a vital equilibrium: high sensitivity (74%-85%) is prioritized for this critical condition where missed cases carry severe consequences, while sufficient specificity (> 76%) is maintained to limit alert fatigue and resource expenditure. The model’s high, stable AUC values (0.82–0.93) across all validations provide further evidence for its excellent performance. We therefore establish 0.54 as a robust default threshold. For optimal local implementation, we suggest that institutions perform calibration within the 0.51–0.56 band, guided by their specific resource constraints and cost-benefit analyses.

Beyond statistical validation, the ultimate value of prediction model lies in its clinical actionability—the ability to trigger timely interventions. Recent evidence underscores that an artificial intelligence (AI)-driven high-risk alert should function not merely as a standalone metric, but as a clinical trigger for a “golden window” of intervention [59]. In our proposed stratified pathway, a high-risk prediction (threshold ≥ 0.54) warrants early specific actions, such as enhanced monitoring (e.g., repeating coagulation profiles and Thromboelastography [TEG]/ Rotational Thromboelastometry [ROTEM] assessment), ruling out reversible causes, and optimizing prophylactic anticoagulation or blood product transfusion strategies. Such a structured response ensures that statistical accuracy is effectively converted into tangible improvements in patient survival.

Several inherent limitations of retrospective validation must be acknowledged in this study. First, although we implemented strict temporal separation and multi-center external validation, the retrospective design cannot fully simulate the complexities of real-world clinical workflows, such as data acquisition delays, dynamic decision-making interference, and potential label drift. Recent independent evaluations have cautioned that AI models deployed solely based on retrospective performance metrics may exhibit limited clinical utility in real-world settings [60]. Consequently, high retrospective performance does not inherently translate into tangible clinical benefit. As emphasized by the American Heart Association (AHA) scientific statement and recent guidelines, the transition from an AI model to a clinical decision-making tool requires rigorous, prospective, and outcome-based evaluation [61–63]. Our findings should therefore be interpreted as hypothesis-generating. Future prospective pragmatic trials are essential to assess the actual impact of this tool on physician behavior and patient survival outcomes.

Second, the lead-time analysis highlights the intrinsic challenges of transitioning from “early recognition” to “prospective prediction”. The performance attenuation observed in validation cohorts, particularly eICU and MIMIC-IV 3.1, is primarily driven by exceptionally high exclusion rates. This reflects a clinical reality where physiological signals often emerge in close temporal proximity to SIC onset, leaving insufficient predictive markers in the remaining subsets after excluding early-onset cases. The rapid progression of sepsis-induced coagulopathy makes long-term forecasting inherently challenging. Nevertheless, the sustained AUC in the Zhongda Hospital cohort confirms the model’s significant stratification and early-warning value in real-world clinical environments. While this model provides substantial incremental value over traditional scoring systems, future research should incorporate higher-frequency dynamic data to address the limited warning window caused by the rapid evolution of the disease.

Third, the use of SHAP for model transparency must be interpreted with caution due to its inherent limitations. While SHAP analysis provides post-hoc feature attribution, it serves as a critical bridge for translating “black-box” mathematical logic into clinical language. As demonstrated in a recent AI implementation study, visualizing risk contributions allows clinicians to interpret al.gorithmic outputs as physiologically meaningful signals [59]. In our model, the high SHAP attribution of PLT and INR reflects early-stage platelet and procoagulant activation, transforming abstract risk scores into a plausible medical narrative that clinicians can intuitively trust.

Crucially, however, these attributions should not be conflated with mechanistic causality. SHAP reflects the internal mathematical logic of the XGBoost algorithm within this specific retrospective cohort rather than biological causality. As highlighted in recent literature, post-hoc explanations in high-stakes healthcare settings may offer a “false sense of interpretability” without providing actionable causal pathways [64]. Furthermore, given the high collinearity common among ICU physiological variables, SHAP attributions are sensitive to feature dependencies, which can lead to unstable or misleading importance rankings [65]. Consequently, our SHAP analyses should be regarded as exploratory tools for understanding model behavior rather than definitive clinical guidelines or a substitute for mechanistic understanding [66]. Future research should explore the development of inherently more interpretable modeling frameworks that better incorporate clinical domain expertise.

## Conclusions

In conclusion, we have established a robust XGBoost model for early SIC prediction through multicenter retrospective validation. This study serves as a vital proof-of-concept and baseline for real-time clinical warning tools. Transitioning to clinical implementation and confirming the model’s ability to enhance patient outcomes remains an essential and urgent priority for future research.

## Supplementary Information

Below is the link to the electronic supplementary material.

Supplementary Material 1

Supplementary Material 2

Supplementary Material 3

## Data Availability

The data that support the findings of this study and key analysis code are available on reasonable request from the corresponding authors. In addition, the implementation code for the Streamlit web application and the data preprocessing pipeline are publicly available in the GitHub repository: https://github.com/Melinda0718/early-SIC-prediction-Webpage.

## Abbreviations

PLT: Platelet count
INR: International Normalized Ratio
SOFA: Sequential Organ Failure Assessment
ML: Machine learning
SHAP: Shapley Additive Explanations
MIMIC-IV: Medical Information Mart for Intensive Care IV database
ICU: Intensive Care Unit
eICU-CRD: eICU Collaborative Research Database
ICD: International Classification of Diseases
CITI: Collaborative Institutional Training Initiative
TRIPOD: Transparent Reporting of a multivariable prediction model for Individual Prognosis or Diagnosis
STROBE: Strengthening the Reporting of Observational Studies in Epidemiology
CKD: Chronic kidney disease
MV: Mechanical ventilation
CRRT: Continuous renal replacement therapy
MCAR: Missing Completely at Random
LRTs: Logistic regression likelihood ratio tests
MAR: Missing at Random
MNAR: Missing Not at Random
RF: Random Forest
XGBoost: Extreme Gradient Boosting'
LightGBM: Light Gradient Boosting Machine
LR: Logistic Regression
SVM: Support Vector Machine
ANOVA: Analysis of Variance
RFECV: Recursive Feature Elimination with Cross-Validation
AUROC: Area under the receiver operating characteristic curve
SMOTE: Synthetic Minority Over-sampling Technique
AUC: Area under the curve
IQR: Interquartile ranges
AUPRC: Area under the precision-recall curve
PPV: Positive predictive value
NPV: Negative predictive value
PR: Precision-recall
DCA: Decision curve analysis
GBDT: Gradient Boosted Decision Trees
PCT: Plateletcrit
FIB: Fibrinogen
FDP: Fibrin degradation products
AI: Artificial Intelligence
TEG: Thromboelastography
ROTEM: Rotational Thromboelastometry
AHA: American Heart Association
