# Supplementary material for: Interpretable machine learning for early prediction of sepsis-induced coagulopathy: a multicenter retrospective development and validation study
Source: BMC Med Inform Decis Mak. 2026 Apr 14;26:183. doi: 10.1186/s12911-026-03471-8 (PMC13188809; doi:10.1186/s12911-026-03471-8)
Supplement: Supplementary file 2 — Supplementary Material 2 [file 12911_2026_3471_MOESM2_ESM.docx]

**Supplementary File 1**

**Interpretable machine learning for early prediction of Sepsis-Induced Coagulopathy: a multicenter retrospective development and validation study**

Qingyun Peng^1†^, Yanzi Guo^1†^, Haoyuan Tang^1^, Shuai Liu^1^, Wei Huang^1^, Xinlong Chen^2^, Shijia Zhong^1^, Zeyuan Zhao^1^, Haofei Wang^1^, Wenhan Hu^1^, Shuhe Yang^1^, Jianfeng Xie^1^, Ming Xue^1^, Shuyuan Qian^1^, Xiaojing Wu^1*^, Yingzi Huang^1**^

1.Jiangsu Provincial Key Laboratory of Critical Care Medicine, Department of Critical Care Medicine, Zhongda Hospital, School of Medicine, Southeast University, Nanjing, 210009, China

2.Department of Critical Care Medicine, Affiliated Hospital of Nantong University, Nantong, 226001, Jiangsu, China.

**Table of Contents**

**Supplementary Methods A:** Definition of “Sepsis”

**Supplementary Methods B:** Sensitivity Analyses

**Supplementary Table S1.** Clinical characteristics between the SIC and non-SIC groups in the derivation cohort and external validation cohorts.

**Supplementary Table S2.** Variables with the percentage of missing data in the derivation and external validation datasets.

**Supplementary Table S3.** Comparison of feature selection methods and performance metrics.

**Supplementary Table S4.** Comparative calibration metrics of machine learning models for early SIC risk stratification in derivation and validation sets.

**Supplementary Table S5.** Model performance for different machine learning models in internal validation dataset for early-stage SIC onset prediction.

**Supplementary Table S6.** Model performance for different machine learning models in internal test dataset for early-stage SIC onset prediction.

**Supplementary Table S7.** Model performance for different machine learning models in external validation datasets for early-stage SIC onset prediction.

**Supplementary Table S8.** Incremental predictive value of the Early-SIC model compared to baseline clinical features and the Day 1 SIC score.

**Supplementary Table S9.** Performance of final model in predicting late-stage SIC onset (Days 4-7) in internal and external validation datasets.

**Supplementary Table S10.** Quantitative assessment of Mean Risk Difference (MRD) in model outputs stratified by clinical subgroups across four datasets.

**Supplementary Table S11.** Sensitivity analysis of model performance: comparison of AUC between the full cohort and non-exposed cohorts across multiple clinical variables.

**Supplementary Table S12.** Subgroup analysis of model discriminative performance stratified by therapeutic interventions, medical history, and baseline organ dysfunction across four cohorts.

**Supplementary Table S13.** Lead time analysis of the Early-SIC model performance after excluding early-onset cases (Day 1 SIC).

**Supplementary Table S14.** Predictive thresholds and performance of the final model across different datasets.

**Supplementary Figure S1.** Density Distribution Comparison Before and After Imputation in the Derivation Cohort.

**Supplementary Figure S2.** The weights of each feature incorporated in different feature selection methods.

**Supplementary Figure S3.** Collinearity analysis in feature selection.

**Supplementary Figure S4.** Comparison of density distributions for selected clinical features between the Derivation set and External validation set.

**Supplementary Figure S5.** The performance and comparison of five different predictive models in internal test set.

**Supplementary Figure S6.** Comparison of Receiver Operating Characteristic (ROC) Curves Based on the Predicted Scores of the XGBoost Model and Benchmark Clinical Indicators or Scores.

**Supplementary Figure S7.** Comprehensive performance evaluation and incremental value analysis of the Early-SIC model across multiple datasets.

**Supplementary Figure S8.** Sensitivity analysis of model-predicted risk scores using Mean Risk Difference (MRD) across therapeutic interventions, medical history, and data missingness patterns.

**Supplementary Figure S9.** Proposed clinical integration framework and actionable management pathway for early sepsis-induced coagulopathy (SIC) prediction.

**Supplementary Methods:**

1. **Definition of “Sepsis”**

According to the Sepsis-3 criteria, sepsis is defined as suspected infection plus an acute increase in the Sequential Organ Failure Assessment (SOFA) score by ≥2 points [1]. In this study, we operationalized sepsis based on established methodologies [2-6], following a three-step process:

**Defining Suspected Infection:** Suspected infection was defined by the co-occurrence of antibiotic administration and body fluid culture sampling. Specifically, if intravenous antibiotics were administered first, a microbiological culture had to be obtained within the subsequent 24 hours. If a culture was obtained first, intravenous antibiotics had to be initiated within the subsequent 72 hours.

**Defining Acute Organ Dysfunction:** Organ dysfunction was defined as an acute increase in the SOFA score by ≥2 points from baseline. For the MIMIC-IV database, we assumed a pre-ICU admission baseline SOFA score of zero.

**Determining Sepsis Onset Time and Final Inclusion:** The sepsis onset time was defined as the earlier of the "suspected infection time" and the "SOFA score deterioration time," provided that the SOFA increase occurred within a window from 48 hours before to 24 hours after the suspected infection time. Patients not meeting this temporal relationship were excluded.

Furthermore, adaptations were made for specific database characteristics:

Due to the inherent characteristics of the eICU-CRD as a multicenter database—specifically the high missingness of microbiological data and the significant variation in the completeness of SOFA score variables across different hospitals—we employed a clinical diagnosis-based approach (utilizing APACHE IV diagnostic codes for 'Sepsis' and its subcategories) to identify septic patients [7]. This methodology has been validated in large-scale electronic health record databases as a robust and clinically relevant alternative. It effectively mitigates selection bias resulting from data missingness and provides a more accurate reflection of the sepsis population as diagnosed by clinicians in real-world practice [8].

In the Zhongda Hospital single-center cohort, the sepsis diagnosis was clinically determined by physicians in accordance with the Sepsis-3.0 criteria as defined by the SSC guidelines [9].

To ensure the highest level of transparency and reproducibility, we hereby report:

Code Source and Consistency: For the MIMIC-IV and eICU-CRD databases, we utilized standardized concept definition views from the official MIT-LCP code repositories (mimic-code, eicu-code). This ensures that our cohort selection rigorously adheres to the Sepsis-3 operational logic described above.

Code Availability: All Structured Query Language (SQL) code used for data extraction and the implementation of the above definitions has been made publicly available. The specific paths are:

MIMIC-IV: https://github.com/MIT-LCP/mimic-code/blob/main/mimic-iv/concepts_postgres/sepsis/suspicion_of_infection.sql

eICU-CRD: https://github.com/MIT-LCP/eicu-code/blob/main/concepts/diagnosis/apache-groups.sql

1. **Sensitivity Analyses**

To rigorously validate the robustness of the Early-SIC prediction model and ensure its clinical reliability across heterogeneous patient populations, we conducted multi-dimensional sensitivity analyses:

**1. Impact of Baseline Comorbidities:** To address potential confounding effects where chronic organ dysfunction might influence the calculation of acute SOFA elevations—thereby affecting the diagnosis of sepsis—we performed sensitivity analyses specifically for patients with a history of chronic kidney disease (CKD), hypertension, and diabetes. Mean Risk Difference (MRD) was employed to quantify the model's capability in distinguishing chronic physiological baseline elevations from acute, sepsis-mediated insults. Furthermore, the predictive performance was re-evaluated in cohorts excluding these relevant baseline comorbidities and compared with the full cohort to assess the model's stability.

**2. Assessment of Data Missingness:** In the derivation cohort, the missing rate for bilirubin was 38.7%. Since bilirubin is essential for calculating the liver SOFA sub-score, a high proportion of missingness could potentially impact the total SOFA score and subsequent SIC diagnosis. In our primary analysis, missing bilirubin values were interpreted as the absence of known severe hepatic dysfunction on that calendar day, and the liver sub-score was consequently assigned a value of 0 [10,11]. To evaluate whether this handling of missing values influenced the results, we used MRD to quantify the difference in average risk between the missing and non-missing groups. Additionally, we compared the predicted risk outputs of the full cohort with those of the complete-case cohort (without missing values) to detect any potential systematic predictive bias.

**3. Evaluation of Clinical Interventions:** To confirm that the model's predictions were driven by intrinsic biological signals rather than being dependent on clinician intervention patterns, we evaluated its discriminative performance and risk score distributions (via MRD) across subgroups stratified by the use of heparin, mechanical ventilation, or continuous renal replacement therapy (CRRT).

**4. Lead-time Analysis:** To verify that the model provides a true "prediction of future risk" rather than merely "early recognition" of existing SIC, we conducted a lead-time analysis. We excluded all patients who already met the SIC criteria within the first 24 hours (Day 1) of ICU admission. The model’s predictive performance was then independently reassessed within this "Day 1 SIC-free" cohort to validate its prospective predictive utility in the absence of pre-existing conditions.

**Reference:**

1. Singer M, Deutschman CS, Seymour CW, Shankar-Hari M, Annane D, Bauer M, et al. The Third International Consensus Definitions for Sepsis and Septic Shock (Sepsis-3). JAMA. 2016;315(8):801-810.

2. Hu W, Chen H, Ma C, Sun Q, Yang M, Wang H, Peng Q, Wang J, Zhang C, Huang W, Xie J, Huang Y. Identification of indications for albumin administration in septic patients with liver cirrhosis. Crit Care. 2023 Jul 28;27(1):300.

3. Kamaleswaran R, Lian J, Lin DL, et al. Predicting volume responsiveness among sepsis patients using clinical data and continuous physiological waveforms. AMIA Annu Symp Proc. 2020; 2020:619–28.

4. Reyna MA, Josef CS, Jeter R, et al. Early prediction of sepsis from clinical data: The PhysioNet/computing in cardiology challenge 2019. Crit Care Med. 2020;48(2):210–7.

5. Raith EP, Udy AA, Bailey M, et al. Australian and New Zealand Intensive Care Society (ANZICS) Centre for Outcomes and Resource Evaluation (CORE). Prognostic Accuracy of the SOFA Score, SIRS Criteria, and qSOFA Score for in‑hospital mortality among adults with suspected infection admitted to the intensive care unit. JAMA. 2017;317(3):290–300.

6. Seymour CW, Liu VX, Iwashyna TJ, Brunkhorst FM, Rea TD, Scherag A, et al. Assessment of clinical criteria for Sepsis: for the third international consensus definitions for sepsis and septic shock (Sepsis-3). JAMA. (2016) 315:762–74.

7. Pollard TJ, Johnson AEW, Raffa JD, Celi LA, Mark RG, Badawi O. The eICU Collaborative Research Database, a freely available multi-center database for critical care research. Sci Data. 2018 Sep 11; 5:180178.

8. Johnson AEW, Aboab J, Raffa JD, Pollard TJ, Deliberato RO, Celi LA, Stone DJ. A Comparative Analysis of Sepsis Identification Methods in an Electronic Database. Crit Care Med. 2018 Apr;46(4):494-499.

9. Evans L, Rhodes A, Alhazzani W, Antonelli M, Coopersmith CM, French C, et al. Surviving sepsis campaign: international guidelines for management of sepsis and septic shock 2021. Intensive Care Med. 2021 Nov;47(11):1181-1247.

10. Brinton DL, Ford DW, Martin RH, Simpson KN, Goodwin AJ, Simpson AN. Missing data methods for intensive care unit SOFA scores in electronic health records studies: results from a Monte Carlo simulation. J Comp Eff Res. 2022;11(1):47-56.

11.Moreno R, Rhodes A, Piquilloud L, Hernandez G, Takala J, Gershengorn HB, et al. The Sequential Organ Failure Assessment (SOFA) Score: has the time come for an update? Crit Care. 2023;27(1):15.

**Table S1. Clinical characteristics between the SIC and non-SIC groups in the derivation cohort and external validation cohorts.**

| Cohort  Variables | Derivation cohort (MIMIC-IV 2.2) | | | External validation cohort 1 (MIMIC-IV 3.1) | | | External validation cohort 2 (eICU) | | | External validation cohort 3 (Zhongda Hospital) | | |
| --- | --- | --- | --- | --- | --- | --- | --- | --- | --- | --- | --- | --- |
|  | NSIC (n=4793) | SIC (n=6240) | *p-value** | NSIC (n=722) | SIC (n=649) | *p-value** | NSIC (n=774) | SIC (n=2074) | *p-value** | NSIC (n=573) | SIC (n=780) | *p-value** |
| **Demographics** |  |  |  |  |  |  |  |  |  |  |  |  |
| Age (years) | 67 (54, 79) | 70 (58, 80) | <0.001 | 65 (55, 75) | 67 (55, 76) | 0.082 | 66 (56, 76) | 67 (56, 77) | 0.442 | 70 (59, 80) | 68 (55, 77) | 0.001 |
| Gender (Male, %) | 2531 (52.8) | 3790 (60.7) | <0.001 | 308 (42.7) | 230 (35.4) | 0.007 | 416 (53.7) | 1102 (53.1) | 0.803 | 401 (70) | 508 (65.1) | 0.069 |
| Weight (kg) | 79 (65, 95) | 80 (68, 95) | 0.003 | 83 (70, 100) | 81 (69, 95) | 0.072 | 80 (65, 100) | 81 (67, 99) | 0.344 | 65 (60, 75) | 65 (60, 70) | 0.195 |
| Height (cm) | 168 (160, 178) | 170 (163, 178) | <0.001 | 168 (163, 178) | 170 (163, 178) | 0.04 | 170 (160, 178) | 170 (162, 178) | 0.621 | 170 (160, 172) | 170 (160, 172) | 0.206 |
| BMI (kg/m²) | 27.9 (23.9, 33.2) | 27.7 (24.1, 32.3) | 0.406 | 29.2 (24.7, 35.1) | 27.9 (24.2, 32.9) | 0.006 | 27.7 (23.3, 33.4) | 28.1 (23.3, 34) | 0.624 | 25.6 (21.2, 32.1) | 25.8 (21.1, 30.2) | 0.238 |
| **Disease severity scores (Median (IQR))** | | | |  |  |  |  |  |  |  |  |  |
| SOFA | 4 (2, 6) | 7 (5, 9) | <0.001 | 6 (4, 8) | 8 (6, 11) | <0.001 | 7 (5, 9) | 9 (6, 11) | <0.001 | 7 (5, 9) | 9 (7, 12) | <0.001 |
| SOFA of respiratory | 2 (0, 3) | 3 (0, 3) | <0.001 | 3 (2, 4) | 3 (2, 4) | 0.596 | 3 (1, 4) | 3 (1, 4) | 0.644 | 2 (0, 3) | 2 (0, 3) | 0.015 |
| SOFA of circulation | 1 (1, 1) | 1 (1, 4) | <0.001 | 1 (1, 3) | 1 (1, 4) | <0.001 | 1 (1, 1) | 1 (1, 1) | <0.001 | 4 (0, 4) | 4 (3, 4) | <0.001 |
| SOFA of coagulation | 0 (0, 0) | 1 (0, 2) | <0.001 | 0 (0, 0) | 1 (0, 2) | <0.001 | 0 (0, 0) | 1 (0, 2) | <0.001 | 0 (0, 0) | 2 (0, 2) | <0.001 |
| SOFA of CNS | 0 (0, 2) | 0 (0, 1) | <0.001 | 0 (0, 1) | 0 (0, 1) | 0.712 | 1 (0, 1) | 1 (0, 1) | 0.885 | 0 (0, 3) | 0 (0, 3) | 0.81 |
| SOFA of liver | 0 (0, 0) | 0 (0, 2) | <0.001 | 0 (0, 0) | 0 (0, 1) | <0.001 | 0 (0, 0) | 0 (0, 2) | <0.001 | 0 (0, 0) | 0 (0, 2) | <0.001 |
| SOFA of renal | 0 (0, 1) | 1 (0, 2) | <0.001 | 1 (0, 2) | 1 (0, 2) | <0.001 | 1 (0, 3) | 2 (1, 3) | <0.001 | 0 (0, 2) | 1 (0, 2) | <0.001 |
| **Laboratory tests (Median (IQR))** | | | |  |  |  |  |  |  |  |  |  |
| Hb(g/dL) | 10.7 (9.1, 12.2) | 9.3 (8, 10.8) | <0.001 | 10.9 (9.3, 12.5) | 9.3 (7.7, 11.2) | <0.001 | 10.7 (9.4, 12.3) | 10.3 (8.9, 12) | <0.001 | 11.1 (9.5, 12.5) | 10.5 (8.8, 11.9) | <0.001 |
| MCH (pg) | 29.9 (28.5, 31.3) | 30 (28.5, 31.3) | 0.750 | 29.4 (28, 30.8) | 29.8 (28.3, 31.2) | 0.003 | 29.6 (27.6, 31) | 29.9 (28, 31.7) | <0.001 | 30 (28.8, 31.2) | 30.3 (29.2, 31.5) | 0.004 |
| WBC (10^9^/L) | 13.9 (10.4, 18.4) | 14.7 (10.5, 19.8) | <0.001 | 14 (10.1, 18.8) | 15.8 (10.6, 21.3) | <0.001 | 14.7 (10.9, 19.3) | 13.6 (8.8, 19.3) | <0.001 | 11.5 (7.9, 16.6) | 11.2 (6.8, 11.9) | 0.509 |
| PLT (10^9^/L) | 204 (165, 259) | 127 (91, 177) | <0.001 | 210 (169, 269) | 128 (85, 184) | <0.001 | 226 (175, 299) | 153 (91, 236) | <0.001 | 196 (158, 257) | 99 (61, 147) | <0.001 |
| NEU count (10^9^/L) | 10.6 (7.4, 14.9) | 10.4 (6.7, 14.9) | 0.01 | 10.4 (7, 14.9) | 10.8 (7.2, 16.4) | 0.193 | - | - | - | 10 (6.7, 14.9) | 10 (5.7, 15.2) | 0.587 |
| LYM count(10^9^/L) | 1.1 (0.7, 1.6) | 1 (0.6, 1.6) | <0.001 | 1 (0.6, 1.4) | 0.8 (0.5, 1.4) | 0.004 | 0.8 (0.5, 1.3) | 0.7 (0.4, 1.2) | 0.065 | 0.6 (0.4, 0.9) | 0.6 (0.3, 0.9) | 0.243 |
| MON count(10^9^/L) | 0.5 (0.3, 0.8) | 0.5 (0.2, 0.8) | <0.001 | 0.6 (0.4, 1.0) | 0.6 (0.3, 1.0) | 0.029 | 0.6 (0.4, 0.8) | 0.6 (0.4, 0.8) | 0.577 | 0.4 (0.3, 0.7) | 0.4 (0.2, 0.7) | 0.026 |
| INR | 1.2 (1.1, 1.2) | 1.5 (1.4, 1.9) | <0.001 | 1.2 (1.1, 1.3) | 1.6 (1.5, 2) | <0.001 | 1.2 (1.1, 1.4) | 2 (1.6, 3) | <0.001 | 1.2 (1.1, 1.3) | 1.5 (1.3, 1.7) | <0.001 |
| PT (seconds) | 12.9 (12, 13.9) | 16.9 (15.2, 20.9) | <0.001 | 13.1 (12.3, 14.1) | 17.7 (15.8, 21.8) | <0.001 | 14.7 (13.1, 16.1) | 22.3 (18.1, 30.9) | <0.001 | 12.8 (11.8, 13.8) | 15.8 (14.2, 17.9) | <0.001 |
| PTT (seconds) | 29.4 (26.4, 35.1) | 37.3 (31.2, 53.3) | <0.001 | 30.4 (27.4, 38.7) | 38.8 (31.4, 69.5) | <0.001 | 32.3 (28.6, 39.5) | 40 (34, 49.2) | <0.001 | 30.7 (27.7, 33.9) | 35 (30.7, 41.6) | <0.001 |
| FIB (g/L) | 2.9 (2.2, 3.8) | 2.1 (1.6, 3.1) | <0.001 | 4.7 (3.3, 6.5) | 2.6 (1.7, 4.8) | <0.001 | 5 (3.5, 6.6) | 3.9 (2.5, 5.4) | <0.001 | 4.3 (3.5, 5.1) | 4 (2.8, 4.8) | <0.001 |
| D-Dimer (ng/mL) | 1365 (606, 3636) | 3627 (1684, 6266) | 0.272 | 1694 (901, 4715) | 3002 (1259, 7350) | 0.044 | - | - | - | 1580 (534, 4252) | 2583 (908, 6622) | <0.001 |
| Glucose (mmol/L) | 8.5 (6.9, 11.2) | 8.1 (6.6, 10.8) | <0.001 | 9.4 (7.5, 13.2) | 9.7 (7.7, 13) | 0.749 | 7.7 (6.1, 10.3) | 7.3 (5.9, 9.4) | <0.001 | 8.5 (6.8, 12.2) | 8.2 (6.5, 11.4) | 0.058 |
| BUN (mmol/L) | 7.1 (5, 11.4) | 9.3 (6, 15.4) | <0.001 | 8.2 (5.7, 13.9) | 10.7 (6.8, 17.9) | <0.001 | 10 (5.9, 16.6) | 12.5 (8, 19.1) | <0.001 | 9.4 (6, 16.1) | 11.1 (7, 17.9) | <0.001 |
| Creatinine (µmol/L) | 88 (71, 133) | 115(80, 186) | <0.001 | 106 (80, 168) | 133 (88, 212) | <0.001 | 115 (80, 221) | 159 (97, 265) | <0.001 | 90 (61, 165) | 118 (76, 204) | <0.001 |
| TBIL (µmol/L) | 8.6 (5.1, 13.7) | 17.1 (8.6, 37.6) | <0.001 | 8.6 (6.8, 13.7) | 13.7 (8.6, 30.8) | <0.001 | 10.3 (6.8, 17.1) | 17.1 (10.3, 37.6) | <0.001 | 12.7 (8.2, 20) | 20.4 (12.2, 37.8) | <0.001 |
| DBIL (µmol/L) | 8.6 (3.4, 31.1) | 35.1 (15.4, 73.5) | <0.001 | 12.8 (7.4, 41) | 41 (17.1, 73.5) | 0.007 | 5.1 (3.4, 10.3) | 12 (5.1, 35.9) | <0.001 | 6.8 (3.9, 11.6) | 12.6 (7.1, 25.6) | <0.001 |
| ALT (U/L) | 27 (17, 54) | 34 (19, 87) | <0.001 | 29 (18, 55) | 33 (19, 82) | 0.004 | 28 (18, 51) | 32 (19, 70) | <0.001 | 28 (18, 42) | 39 (24, 74.5) | <0.001 |
| AST (U/L) | 39 (24, 75) | 57 (31, 146) | <0.001 | 45 (27, 80) | 63 (31, 137) | <0.001 | 37 (23, 63) | 48 (27, 118) | <0.001 | 36 (24, 61) | 49 (31, 108) | <0.001 |
| ALP (U/L) | 80 (62, 109) | 85 (60, 129) | <0.001 | 80 (62, 105) | 89 (60, 126) | 0.024 | 98 (73, 142) | 104 (75, 154) | 0.029 | 81 (61, 116) | 86 (60, 139) | 0.047 |
| TC (mmol/L) | 4.0 (3.3, 4.8) | 3.1 (2.2, 3.8) | <0.001 | 4.1 (3.4, 4.9) | 2.4 (1.7, 2.7) | <0.001 | 2.7 (2.6, 2.8) | 2.7 (2.6, 2.8) | 0.239 | 2.9 (2.3, 3.8) | 2.2 (1.7, 2.8) | <0.001 |
| TG (mmol/L) | 1.4 (0.7, 2.2) | 1.3 (0.9, 2.3) | 0.609 | 2.2 (1.4, 3.7) | 1.9 (1.1, 3.5) | 0.11 | 1.5 (0.9, 2.4) | 1.3 (1, 2) | 0.521 | 1.4 (0.9, 2.2) | 1.4 (0.9, 2.2) | 0.648 |
| pH | 7.3 (7.3, 7.4) | 7.3 (7.2, 7.4) | <0.001 | 7.3 (7.2, 7.4) | 7.3 (7.2, 7.3) | 0.001 | 7.4 (7.3, 7.4) | 7.3 (7.3, 7.4) | 0.267 | 7.4 (7.3, 7.5) | 7.4 (7.3, 7.4) | 0.053 |
| PaO_2_ (mmHg) | 75 (47, 112) | 72 (44, 100) | <0.001 | 53 (37, 77) | 53 (38, 84) | 0.143 | 76 (61, 100) | 76 (61, 99) | 0.743 | 104 (82, 147) | 103 (80, 148) | 0.805 |
| Lactate (mmol/L) | 1.9 (1.3, 3) | 2.6 (1.8, 4.2) | <0.001 | 2 (1.3, 3.2) | 3.2 (1.8, 5.3) | <0.001 | 2 (1.4, 3) | 2.6 (1.6, 4.2) | <0.001 | 1.5 (1, 2.2) | 2.1 (1.3, 3.5) | <0.001 |
| PaO_2_/FiO_2_ ratio | 171 (98, 283) | 152 (90, 240) | <0.001 | 111 (73.4, 208) | 122 (76, 212) | 0.255 | 122 (83, 181) | 135 (90, 193) | 0.032 | 222 (159, 326) | 234 (158, 330) | 0.429 |
| Bicarbonate (mmol/L) | 23 (21, 26) | 22 (18, 25) | 0.006 | 22 (19, 25) | 20 (18, 24) | 0.014 | 23 (20, 26) | 22 (18, 25) | <0.001 | 21 (19, 25) | 19 (16, 23) | <0.001 |
| Potassium (mmol/L) | 4.2 (3.8, 5) | 4.9 (4.2, 5.5) | <0.001 | 4.2 (3.7, 4.9) | 4.4 (3.8, 5.1) | 0.001 | 4.1 (3.8, 4.6) | 4.1 (3.7, 4.6) | 0.871 | 3.8 (3.5, 4.3) | 3.8 (3.5, 4.2) | 0.351 |
| Sodium (mmol/L) | 138 (135, 140) | 138 (136, 140) | 0.799 | 138 (135, 141) | 136 (133, 140) | <0.001 | 137 (134, 141) | 137 (134, 140) | 0.106 | 137 (134, 141) | 138 (134, 142) | 0.202 |
| **Vital signs (Median (IQR))** | | | |  |  |  |  |  |  |  |  |  |
| Heart rate (bpm) | 104 (91, 119) | 104 (90, 120) | 0.131 | 104 (91, 118) | 105 (92, 122) | 0.059 | 97 (86, 110) | 98 (84, 111) | 0.942 | 90 (80, 102) | 96 (86, 106) | <0.001 |
| MAP (mmHg) | 59 (52, 66) | 56 (50, 62) | <0.001 | 60 (55, 66) | 58 (51, 64) | <0.001 | 78 (70, 85) | 75 (69, 83) | <0.001 | 86 (80, 91) | 86 (81, 91) | 0.88 |
| Temperature (℃) | 37.5 (37.1, 38.1) | 37.4 (37, 38) | <0.001 | 37.6 (37.2, 38.2) | 37.4 (37.1, 38.1) | 0.006 | 37 (36.6, 37.5) | 36.9 (36.5, 37.4) | <0.001 | 37.1 (36.8, 37.5) | 37.1 (36.6, 37.6) | 0.112 |
| Respiratory rate(bpm) | 27 (24, 32) | 27 (24, 32) | 0.321 | 28 (24, 33) | 28 (24, 32) | 0.174 | 22 (19, 26) | 22 (19, 26) | 0.098 | 19 (17, 22) | 20 (17, 22) | 0.391 |
| **Comorbidity diseases (n (%))** | | | |  |  |  |  |  |  |  |  |  |
| Hypertension | 3000 (62.6) | 3894 (62.4) | 0.856 | 421 (58.3) | 370 (57) | 0.666 | 65 (8.4) | 131 (6.3) | 0.062 | 330 (57.6) | 406 (52.1) | 0.049 |
| Diabetes | 1478 (30.8) | 1953 (31.3) | 0.618 | 264 (36.6) | 222 (34.2) | 0.393 | 23 (3) | 40 (1.9) | 0.123 | 190 (33.2) | 239 (30.6) | 0.355 |
| CKD | 867 (18.1) | 1577 (25.3) | <0.001 | 142 (19.7) | 183 (28.2) | <0.001 | 100 (12.9) | 336 (16.2) | 0.035 | 58 (10.1) | 61 (7.8) | 0.168 |
| Chronic liver disease | 15 (0.31) | 52 (0.83) | 0.001 | 6 (0.8) | 4 (0.6) | 0.757 | 0 | 0 | - | 5 (0.9) | 26 (3.3) | 0.005 |
| **Support treatment (n (%))** | | | |  |  |  |  |  |  |  |  |  |
| Mechanical ventilation | 2939 (61.3) | 4358 (69.8) | <0.001 | 667 (92.4) | 588 (90.6) | 0.277 | 220 (28.4) | 587 (28.3) | 0.986 | 249 (43.5) | 452 (57.9) | <0.001 |
| CRRT treatment | 130 (2.7) | 503 (8.1) | <0.001 | 92 (12.7) | 112 (17.3) | 0.023 | 36 (4.7) | 153 (7.4) | 0.018 | 63 (11) | 166 (21.3) | <0.001 |
| Heparin use | 2493 (52) | 2351 (37.7) | <0.001 | 447 (61.9) | 325 (50.1) | <0.001 | 122 (15.9) | 165 (7.9) | <0.001 | 512 (89.4) | 693 (88.8) | 0.835 |
| **Outcome (Median (IQR))** | | | |  |  |  |  |  |  |  |  |  |
| LOS of hospital (days) | 10.7 (6.7, 17.9) | 10.4 (6.6, 17.4) | 0.326 | 16 (8.9, 28) | 14.8 (8.2, 25.9) | 0.123 | 9.3 (5.8, 15.2) | 9.3 (5.8, 15.7) | 0.888 | 20.7 (12.5, 33.1) | 18.1 (10.6, 28.6) | 0.001 |
| LOS of ICU (days) | 4.9 (3.0, 9.5) | 4.4 (2.9, 8.1) | <0.001 | 8.9 (4.7, 16.9) | 6.4 (3.6, 10.9) | <0.001 | 4.4 (2.9, 8.2) | 4.5 (3, 7.7) | 0.825 | 11.6 (5.8, 20.9) | 9.3 (4.9, 16.6) | <0.001 |
| 28-day mortality (%) | 15.8 | 19.3 | <0.001 | 21.7 | 27 | 0.044 | 14.2 | 23.9 | <0.001 | 21.5 | 27.1 | 0.022 |
| ICU mortality (%) | 26.2 | 28.6 | 0.006 | 20 | 24 | 0.078 | 9.6 | 14.8 | <0.001 | 20.4 | 23.2 | 0.248 |
| Hospital mortality (%) | 22.6 | 24.2 | 0.053 | 24.4 | 31.4 | 0.004 | 15.3 | 25.1 | <0.001 | 22.9 | 28.6 | 0.021 |

**Note:** *SIC group compare with NSIC group; - indicates no data for this item.

**Abbreviations:** SIC: Sepsis-induced Coagulopathy; NSIC: Non Sepsis-induced Coagulopathy; MIMIC-IV: Medical Information Mart for Intensive Care IV; eICU: eICU Collaborative Research Database; BMI: Body Mass Index; IQR: Inter Quartile Range; SOFA: Sequential Organ Failure Assessment; CNS: Central Nervous System; Hb: Hemoglobin; MCH: Mean Corpuscular Hemoglobin; WBC: White Blood Cell; PLT: Platelet; NEU：neutrophils; LYM: lymphocytes; MON: monocytes; INR: International Normalized Ratio; PT: Prothrombin Time; PTT: Partial Thromboplastin Time; FIB: Fibrinogen; BUN: Blood Urea Nitrogen; TBIL: total bilirubin; DBIL: direct bilirubin; ALT: Alanine aminotransferase; AST: Aspartate aminotransferase; ALP: Alkaline phosphatase; TC: Total cholesterol; TG: Triglycerides; pH: Potential of Hydrogen; PaO_2_: Partial pressure of oxygen; FiO_2_: Fraction of inspiration oxygen; MAP: Mean Arterial Pressure; CKD: Chronic kidney disease; CRRT: Continuous Renal Replacement Therapy; LOS: Length of hospital Stay; ICU: Intensive Care Unit.

**Table S2. Variables with the percentage of missing data in the derivation and external validation datasets.**

| Variables | Derivation Dataset | Percentage of Missing Data | MIMIC-IV 3.1 | Percentage of Missing Data | eICU | Percentage of Missing Data | Zhongda Hospital | Percentage of Missing Data |
| --- | --- | --- | --- | --- | --- | --- | --- | --- |
| Demographics | | | | | | | | |
| Age | 11033 | 0.0% | 1371 | 0.0% | 2848 | 0.0% | 1353 | 0.0% |
| Gender | 11033 | 0.0% | 1371 | 0.0% | 2848 | 0.0% | 1353 | 0.0% |
| Weight | 10801 | 2.1% | 1348 | 1.7% | 2812 | 1.3% | 1353 | 0.0% |
| Height | 7770 | 29.6% | 1206 | 12.0% | 2838 | 0.4% | 1342 | 0.8% |
| BMI | 7770 | 29.6% | 1206 | 12.0% | 2807 | 1.4% | 1342 | 0.8% |
| Disease severity scores | | | | | | | | |
| SOFA | 11033 | 0.0% | 1371 | 0.0% | 2848 | 0.0% | 1353 | 0.0% |
| SOFA of respiratory | 7727 | 30.0% | 1371 | 0.0% | 2848 | 0.0% | 1353 | 0.0% |
| SOFA of circulation | 11022 | 0.1% | 1371 | 0.0% | 2848 | 0.0% | 1353 | 0.0% |
| SOFA of coagulation | 11030 | 0.0% | 1371 | 0.0% | 2848 | 0.0% | 1353 | 0.0% |
| SOFA of CNS | 11018 | 0.1% | 1371 | 0.0% | 2848 | 0.0% | 1353 | 0.0% |
| SOFA of liver | 6763 | 38.7% | 1371 | 0.0% | 2848 | 0.0% | 1353 | 0.0% |
| SOFA of renal | 11033 | 0.0% | 1371 | 0.0% | 2848 | 0.0% | 1353 | 0.0% |
| Laboratory tests | | | | | | | | |
| Hb | 11030 | 0.0% | 1371 | 0.0% | 2847 | 0.0% | 1352 | 0.1% |
| MCH | 11017 | 0.1% | 1371 | 0.0% | 2721 | 4.5% | 1353 | 0.0% |
| WBC | 11032 | 0.0% | 1371 | 0.0% | 2845 | 0.1% | 1353 | 0.0% |
| Platelet count | 11030 | 0.0% | 1371 | 0.0% | 2848 | 0.0% | 1353 | 0.0% |
| Neutrophil count | 7550 | 31.6% | 1093 | 20.3% | - | - | 1348 | 0.4% |
| Lymphocyte count | 7555 | 31.5% | 1093 | 20.3% | 2317 | 18.6% | 1348 | 0.4% |
| Monocyte count | 7550 | 31.6% | 1093 | 20.3% | 2292 | 19.5% | 1348 | 0.4% |
| INR | 11033 | 0.0% | 1371 | 0.0% | 2848 | 0.0% | 1353 | 0.0% |
| PT | 11033 | 0.0% | 1371 | 0.0% | 2762 | 3.0% | 1353 | 0.0% |
| PTT | 10968 | 0.6% | 1366 | 0.4% | 1960 | 31.2% | 1353 | 0.0% |
| FIB | 4373 | 60.4% | 529 | 61.4% | 423 | 85.1% | 1353 | 0.0% |
| D-Dimer | 30 | 99.7% | 207 | 84.9% | - | - | 1352 | 0.1% |
| Glucose | 10985 | 0.4% | 1370 | 0.1% | 2833 | 0.5% | 1337 | 1.2% |
| BUN | 11030 | 0.0% | 1371 | 0.0% | 2845 | 0.1% | 1272 | 6.0% |
| Creatinine | 11032 | 0.0% | 1371 | 0.0% | 2843 | 0.2% | 1337 | 1.2% |
| Total bilirubin | 6763 | 38.7% | 1371 | 0.0% | 2617 | 8.1% | 1143 | 15.5% |
| Direct bilirubin | 650 | 94.1% | 83 | 93.9% | 757 | 73.4% | 1047 | 22.6% |
| ALT | 6812 | 38.3% | 1346 | 1.8% | 2655 | 6.8% | 1291 | 4.6% |
| AST | 6828 | 38.1% | 1366 | 0.4% | 2661 | 6.6% | 1337 | 1.2% |
| ALP | 6767 | 38.7% | 1363 | 0.6% | 2653 | 6.8% | 1337 | 1.2% |
| Total cholesterol | 276 | 97.5% | 57 | 95.8% | 2845 | 0.1% | 1332 | 1.6% |
| Triglycerides | 469 | 95.7% | 221 | 83.9% | 309 | 89.2% | 1337 | 1.2% |
| pH | 9385 | 14.9% | 1371 | 0.0% | 1766 | 38.0% | 1343 | 0.7% |
| PaO2 | 9385 | 14.9% | 1371 | 0.0% | 1779 | 37.5% | 1343 | 0.7% |
| Lactate | 8602 | 22.0% | 1280 | 6.6% | 2491 | 12.5% | 1343 | 0.7% |
| PaO2/FiO2 ratio | 7727 | 30.0% | 1371 | 0.0% | 1352 | 52.5% | 1343 | 0.7% |
| Bicarbonate | 293 | 97.3% | 185 | 86.5% | 2683 | 5.8% | 1343 | 0.7% |
| Potassium | 5145 | 53.4% | 702 | 48.8% | 2844 | 0.1% | 1343 | 0.7% |
| Sodium | 4392 | 60.2% | 511 | 62.7% | 2845 | 0.1% | 1343 | 0.7% |
| Vital signs | | | | | | | | |
| Heart rate | 11022 | 0.1% | 1371 | 0.0% | 2628 | 7.7% | 1351 | 0.1% |
| MAP | 11022 | 0.1% | 1371 | 0.0% | 924 | 67.6% | 1351 | 0.1% |
| Temperature | 10309 | 6.6% | 1327 | 3.2% | 2728 | 4.2% | 1284 | 5.1% |
| Respiratory rate | 11018 | 0.1% | 1371 | 0.0% | 2591 | 9.0% | 1352 | 0.1% |
| Comorbidity diseases | | | | | | | | |
| Hypertension | 11033 | 0.0% | 1371 | 0.0% | 2848 | 0.0% | 1353 | 0.0% |
| Diabetes | 11033 | 0.0% | 1371 | 0.0% | 2848 | 0.0% | 1353 | 0.0% |
| Chronic kidney disease | 11033 | 0.0% | 1371 | 0.0% | 2848 | 0.0% | 1353 | 0.0% |
| Chronic liver disease | 11033 | 0.0% | 1371 | 0.0% | 2848 | 0.0% | 1353 | 0.0% |
| Support treatment | | | | | | | | |
| Mechanical ventilation | 11033 | 0.0% | 1371 | 0.0% | 2848 | 0.0% | 1353 | 0.0% |
| CRRT treatment | 11033 | 0.0% | 1371 | 0.0% | 2721 | 4.5% | 1353 | 0.0% |

**Note:** - indicates no data for this item.

**Abbreviations:** MIMIC-IV: Medical Information Mart for Intensive Care IV; eICU: eICU Collaborative Research Database; BMI: Body Mass Index; SOFA: Sequential Organ Failure Assessment; CNS: Central Nervous System; Hb: Hemoglobin; MCH: Mean Corpuscular Hemoglobin; WBC: White Blood Cell; INR: International Normalized Ratio; PT: Prothrombin Time; PTT: Partial Thromboplastin Time; FIB: Fibrinogen; BUN: Blood Urea Nitrogen; ALT: Alanine aminotransferase; AST: Aspartate aminotransferase; ALP: Alkaline phosphatase; pH: Potential of Hydrogen; PaO_2_: Partial pressure of oxygen; FiO2:Fraction of inspiration oxygen; MAP: Mean Arterial Pressure; CRRT: Continuous Renal Replacement Therapy; ICU: Intensive Care Unit.

**Table S3. Comparison of feature selection methods and performance metrics.**

| **Feature selection method** | **Selected feature numbers** | **Average PSI** | **AUC of training Set** | **AUC of internal validation set** | **Weighted scores** |
| --- | --- | --- | --- | --- | --- |
| **RFECV+RF** | 10 | 0.218 | 0.781 | 0.789 | 0.200 |
| **RFECV+LR** | 11 | 0.203 | 0.780 | 0.791 | 0.514 |
| **Kruskal-Wallis test** | 11 | 0.199 | 0.780 | 0.791 | 0.394 |
| **Null Importance** | 13 | 0.176 | 0.780 | 0.789 | 0.668 |

**Note:** Feature set selection, integrating stability-weighted selection criteria (60%) and predictive performance (40%) for best practices. Feature set using null importance outperformed others with a feature number of 12.

**Abbreviations:** PSI: Population Stability Index; AUC: Area Under Curve; RFECV: Recursive Feature Elimination with Cross-Validation; RF: Random Forest; LR: Logistic Regression.

**Table S4.** **Comparative calibration metrics of machine learning models for early SIC risk stratification in derivation and validation sets.**

| **Dataset** | **Calibration Model** | **Calibration Slope** | **Calibration Intercept** | **Calibration Function** | **Brier Score** | **ECE** | **MCE** | **Reliability Loss** | **R-squared** |
| --- | --- | --- | --- | --- | --- | --- | --- | --- | --- |
| **Derivation cohort（MIMIC IV 2.2）** | **XGBoost** | 0.9899 | 0.0361 | y=0.9899x+0.0361 | 0.1709 | 0.0497 | 0.0786 | 0.0276 | 0.9724 |
|  | **RF** | 0.9993 | 0.0372 | y=0.9993x+0.0372 | 0.1723 | 0.0373 | 0.0885 | 0.0101 | 0.9899 |
|  | **LightGBM** | 0.9816 | 0.0435 | y=0.9816x+0.0435 | 0.1707 | 0.0482 | 0.0956 | 0.0306 | 0.9694 |
|  | **LR** | 0.9083 | 0.1086 | y=0.9083x+0.1086 | 0.1913 | 0.0766 | 0.2799 | 0.0926 | 0.9074 |
|  | **SVM** | 0.9785 | 0.0462 | y=0.9785x+0.0462 | 0.1747 | 0.0343 | 0.0873 | 0.015 | 0.985 |
| **MIMIC IV 3.1** | **XGBoost** | 0.9813 | 0.0257 | y=0.9813x+0.0257 | 0.1733 | 0.0347 | 0.0923 | 0.0247 | 0.9753 |
|  | **RF** | 0.8521 | 0.1156 | y=0.8521x+0.1156 | 0.1769 | 0.0545 | 0.303 | 0.1095 | 0.8905 |
|  | **LightGBM** | 0.9669 | 0.0346 | y=0.9669x+0.0346 | 0.1734 | 0.0406 | 0.0794 | 0.0287 | 0.9713 |
|  | **LR** | 0.7827 | 0.1811 | y=0.7827x+0.1811 | 0.1902 | 0.0949 | 0.5397 | 0.3127 | 0.6873 |
|  | **SVM** | 0.9495 | 0.0442 | y=0.9495x+0.0442 | 0.1743 | 0.0339 | 0.095 | 0.0278 | 0.9722 |
| **eICU** | **XGBoost** | 1.0813 | 0.0447 | y=1.0813x+0.0447 | 0.1381 | 0.0978 | 0.147 | 0.0309 | 0.9691 |
|  | **RF** | 1.0763 | 0.0593 | y=1.0763x+0.0593 | 0.1468 | 0.1011 | 0.147 | 0.0156 | 0.9844 |
|  | **LightGBM** | 1.0164 | 0.0993 | y=1.0164x+0.0993 | 0.1449 | 0.1083 | 0.1988 | 0.0328 | 0.9672 |
|  | **LR** | 0.8942 | 0.2436 | y=0.8942x+0.2436 | 0.2013 | 0.1906 | 0.3074 | 0.1033 | 0.8967 |
|  | **SVM** | 0.9435 | 0.235 | y=0.9435x+0.2350 | 0.2004 | 0.225 | 0.4107 | 0.2396 | 0.7604 |
| **Zhongda Hospital** | **XGBoost** | 0.7865 | -0.2021 | y=0.7865x-0.2021 | 0.2484 | 0.3189 | 0.4234 | 0.1196 | 0.8804 |
|  | **RF** | 0.8633 | -0.2213 | y=0.8633x-0.2213 | 0.2464 | 0.2961 | 0.3843 | 0.1235 | 0.8765 |
|  | **LightGBM** | 0.751 | -0.1354 | y=0.7510x-0.1354 | 0.2239 | 0.2606 | 0.3959 | 0.093 | 0.907 |
|  | **LR** | 0.8588 | -0.1453 | y=0.8588x-0.1453 | 0.2079 | 0.2162 | 0.3316 | 0.0964 | 0.9036 |
|  | **SVM** | 0.674 | -0.0774 | y=0.6740x-0.0774 | 0.2189 | 0.2416 | 0.375 | 0.053 | 0.947 |

**Abbreviations:** SIC: Sepsis-induced coagulopathy; ECE: Expected Calibration Error; MCE: Maximum Calibration Error; XGBoost: Extreme Gradient Boosting; LightGBM: Light Gradient Boosting Machine; SVM: Support Vector Machine; RF: Random Forest; LR: Logistic Regression; MIMIC-IV: Medical Information Mart for Intensive Care IV; eICU: eICU Collaborative Research Database.

**Table S5. Model performance for different machine learning models in internal validation dataset for early-stage SIC onset prediction.**

| **Machine Learning Models** | **Accuracy** | **F1 Score** | **AUC** | **AUPRC** | **Sensitivity** | **Specificity** | **PPV** | **NPV** |
| --- | --- | --- | --- | --- | --- | --- | --- | --- |
| **XGBoost** | 0.745  (0.727, 0.762) | 0.754  (0.735, 0.772) | 0.827  (0.811, 0.843) | 0.854  (0.836, 0.872) | 0.719  (0.694, 0.744) | 0.776  (0.749, 0.801) | 0.793  (0.769, 0.816) | 0.699  (0.672, 0.726) |
| **Random Forest** | 0.742  (0.723, 0.758) | 0.752  (0.731, 0.770) | 0.823  (0.805, 0.839) | 0.852  (0.833, 0.869) | 0.719  (0.695, 0.744) | 0.769  (0.743, 0.794) | 0.787  (0.763, 0.810) | 0.697  (0.671, 0.723) |
| **LightGBM** | 0.746  (0.729, 0.764) | 0.756  (0.737, 0.774) | 0.827  (0.808, 0.844) | 0.853  (0.834, 0.872) | 0.723  (0.698, 0.747) | 0.774  (0.749, 0.800) | 0.791  (0.767, 0.816) | 0.702  (0.675, 0.728) |
| **Logistics Regression** | 0.712  (0.694, 0.731) | 0.715  (0.693, 0.737) | 0.785  (0.766, 0.803) | 0.821  (0.800, 0.841) | 0.668  (0.641, 0.693) | 0.764  (0.738, 0.789) | 0.771  (0.747, 0.795) | 0.659  (0.634, 0.685) |
| **SVM** | 0.737  (0.718, 0.753) | 0.743  (0.722, 0.761) | 0.817  (0.799, 0.833) | 0.840  (0.819, 0.860) | 0.700  (0.675, 0.726) | 0.780  (0.757, 0.805) | 0.791  (0.769, 0.814) | 0.686  (0.663, 0.713) |

**Abbreviations:** SIC: Sepsis-induced coagulopathy; AUC: Area Under Curve; AUPRC: Area Under the Precision-Recall Curve; PPV: Positive Predictive Value; NPV: Negative Predictive Value; XGBoost: Extreme Gradient Boosting; LightGBM: Light Gradient Boosting Machine; SVM: Support Vector Machine.

**Table S6.** **Model performance for different machine learning models in internal test dataset for early-stage SIC onset prediction.**

| **Machine Learning Models** | **Accuracy** | **F1 Score** | **AUC** | **AUPRC** | **Sensitivity** | **Specificity** | **PPV** | **NPV** |
| --- | --- | --- | --- | --- | --- | --- | --- | --- |
| **XGBoost** | 0.740  (0.713, 0.765) | 0.740  (0.710, 0.768) | 0.820  (0.795, 0.844) | 0.845  (0.816, 0.870) | 0.705  (0.668, 0.743) | 0.778  (0.743, 0.812) | 0.780  (0.746, 0.813) | 0.703  (0.666, 0.740) |
| **Random Forest** | 0.734  (0.709, 0.760) | 0.738  (0.708, 0.767) | 0.810  (0.785, 0.835) | 0.839  (0.811, 0.865) | 0.711  (0.675, 0.746) | 0.760  (0.726, 0.795) | 0.767  (0.732, 0.801) | 0.703  (0.665, 0.738) |
| **LightGBM** | 0.736  (0.711, 0.762) | 0.738  (0.710, 0.769) | 0.821  (0.796, 0.846) | 0.845  (0.817, 0.873) | 0.705  (0.669, 0.740) | 0.771  (0.734, 0.807) | 0.774  (0.742, 0.813) | 0.701  (0.662, 0.737) |
| **Logistics Regression** | 0.726  (0.699, 0.751) | 0.723  (0.693, 0.750) | 0.789  (0.760, 0.813) | 0.809  (0.776, 0.840) | 0.677  (0.637, 0.713) | 0.782  (0.746, 0.815) | 0.776  (0.738, 0.810) | 0.685  (0.650, 0.721) |
| **SVM** | 0.740  (0.712, 0.767) | 0.739  (0.707, 0.766) | 0.818  (0.792, 0.842) | 0.844  (0.816, 0.870) | 0.697  (0.660, 0.731) | 0.789  (0.753, 0.824) | 0.786  (0.750, 0.822) | 0.700  (0.662, 0.736) |

**Abbreviations:** SIC: Sepsis-induced coagulopathy; AUC: Area Under Curve; AUPRC: Area Under the Precision-Recall Curve; PPV: Positive Predictive Value; NPV: Negative Predictive Value; XGBoost: Extreme Gradient Boosting; LightGBM: Light Gradient Boosting Machine; SVM: Support Vector Machine.

**Table S7. Model performance for different machine learning models in external validation datasets for early-stage SIC onset prediction.**

| **Dataset** | **Machine Learning Models** | **Accuracy** | **F1 Score** | **AUC** | **AUPRC** | **Sensitivity** | **Specificity** | **PPV** | **NPV** |
| --- | --- | --- | --- | --- | --- | --- | --- | --- | --- |
| **MIMIC IV 3.1** | **XGBoost** | 0.853  (0.837,  0.870) | 0.872  (0.855,  0.887) | 0.927  (0.916,  0.939) | 0.954  (0.946,  0.962) | 0.856  (0.834,  0.878) | 0.850  (0.826,  0.875) | 0.889  (0.870,  0.908) | 0.808  (0.779,  0.838) |
|  | **RF** | 0.829  (0.811,  0.846) | 0.844  (0.826,  0.860) | 0.909  (0.895,  0.921) | 0.941  (0.930,  0.950) | 0.792  (0.769,  0.817) | 0.880  (0.855,  0.902) | 0.903  (0.883,  0.921) | 0.751  (0.724,  0.780) |
|  | **LightGBM** | 0.830  (0.811,  0.847) | 0.843  (0.825,  0.860) | 0.917  (0.905,  0.928) | 0.947  (0.938,  0.955) | 0.784  (0.760,  0.807) | 0.894  (0.871,  0.915) | 0.912  (0.893,  0.929) | 0.747  (0.717,  0.776) |
|  | **LR** | 0.819  (0.801,  0.837) | 0.829  (0.811,  0.848) | 0.912  (0.899,  0.925) | 0.941  (0.931,  0.951) | 0.751  (0.724,  0.777) | 0.915  (0.895,  0.935) | 0.926  (0.907,  0.943) | 0.724  (0.695,  0.754) |
|  | **SVM** | 0.772  (0.752,  0.790) | 0.796  (0.776,  0.815) | 0.843  (0.825,  0.860) | 0.886  (0.868,  0.903) | 0.762  (0.735,  0.789) | 0.785  (0.757,  0.815) | 0.833  (0.810,  0.857) | 0.702  (0.670,  0.732) |
| **eICU** | **XGBoost** | 0.809  (0.795,  0.823) | 0.871  (0.861,  0.882) | 0.859  (0.845,  0.873) | 0.942  (0.933,  0.949) | 0.885  (0.871,  0.898) | 0.600  (0.563,  0.631) | 0.859  (0.843,  0.873) | 0.654  (0.619,  0.687) |
|  | **RF** | 0.787  (0.772,  0.801) | 0.850  (0.839,  0.861) | 0.846  (0.831,  0.862) | 0.936  (0.927,  0.944) | 0.826  (0.809,  0.843) | 0.678  (0.646,  0.710) | 0.876  (0.862,  0.891) | 0.587  (0.552,  0.620) |
|  | **LightGBM** | 0.783  (0.769,  0.797) | 0.846  (0.834,  0.857) | 0.852  (0.836,  0.866) | 0.939  (0.931,  0.947) | 0.810  (0.792,  0.826) | 0.711  (0.678,  0.742) | 0.885  (0.870,  0.899) | 0.576  (0.543,  0.607) |
|  | **LR** | 0.715  (0.700,  0.731) | 0.779  (0.763,  0.792) | 0.810  (0.792,  0.828) | 0.905  (0.891,  0.920) | 0.683  (0.666,  0.704) | 0.801  (0.773,  0.828) | 0.904  (0.891,  0.918) | 0.480  (0.453,  0.507) |
|  | **SVM** | 0.689  (0.671,  0.707) | 0.745  (0.727,  0.761) | 0.841  (0.826,  0.857) | 0.928  (0.918,  0.938) | 0.619  (0.599,  0.640) | 0.879  (0.856,  0.902) | 0.934  (0.921,  0.947) | 0.457  (0.432,  0.483) |
| **Zhongda Hospital** | **XGBoost** | 0.630  (0.603,  0.656) | 0.571  (0.537,  0.606) | 0.825  (0.801,  0.846) | 0.590  (0.535,  0.645) | 0.931  (0.906,  0.958) | 0.522  (0.491,  0.556) | 0.412  (0.377,  0.445) | 0.955  (0.938,  0.972) |
|  | **RF** | 0.629  (0.601,  0.654) | 0.569  (0.532,  0.603) | 0.819  (0.793,  0.844) | 0.592  (0.538,  0.649) | 0.925  (0.898,  0.951) | 0.523  (0.491,  0.557) | 0.411  (0.377,  0.446) | 0.951  (0.932,  0.968) |
|  | **LightGBM** | 0.673  (0.647,  0.699) | 0.594  (0.558,  0.629) | 0.824  (0.799,  0.848) | 0.586  (0.530,  0.644) | 0.905  (0.871,  0.936) | 0.590  (0.559,  0.619) | 0.442  (0.407,  0.479) | 0.945  (0.925,  0.964) |
|  | **LR** | 0.663  (0.637,  0.687) | 0.583  (0.546,  0.616) | 0.818  (0.795,  0.844) | 0.586  (0.536,  0.645) | 0.890  (0.855,  0.924) | 0.582  (0.549,  0.613) | 0.433  (0.395,  0.470) | 0.936  (0.915,  0.956) |
|  | **SVM** | 0.702  (0.676,  0.727) | 0.600  (0.560,  0.637) | 0.803  (0.778,  0.829) | 0.535  (0.482,  0.597) | 0.845  (0.809,  0.882) | 0.651  (0.620,  0.681) | 0.465  (0.427,  0.504) | 0.922  (0.901,  0.941) |

**Abbreviations:** SIC: Sepsis-induced coagulopathy; AUC: Area Under Curve; AUPRC: Area Under the Precision-Recall Curve; PPV: Positive Predictive Value; NPV: Negative Predictive Value; XGBoost: Extreme Gradient Boosting; LightGBM: Light Gradient Boosting Machine; SVM: Support Vector Machine; RF: Random Forest; LR: Logistic Regression; MIMIC-IV: Medical Information Mart for Intensive Care IV; eICU: eICU Collaborative Research Database.

**Table S8. Incremental predictive value of the Early-SIC model compared to baseline clinical features and the Day 1 SIC score.**

| **Dataset** | **Feature** | **Baseline AUC** | **Early-SIC AUC** | **ΔAUC** | **Relative Improvement (%)** | ***P value*** |
| --- | --- | --- | --- | --- | --- | --- |
| **MIMIC-IV 2.2** | **SOFA circulation** | 0.6308 | 0.8193 | 0.1884 | 29.87 | <0.001 |
|  | **SOFA renal** | 0.622 | 0.8193 | 0.1972 | 31.71 | <0.001 |
|  | **Platelet Count** | 0.7159 | 0.8193 | 0.1034 | 14.44 | <0.001 |
|  | **INR** | 0.7295 | 0.8193 | 0.0897 | 12.3 | <0.001 |
|  | **SOFA respiratory** | 0.5441 | 0.8193 | 0.2751 | 50.56 | <0.001 |
|  | **Lactate** | 0.6362 | 0.8193 | 0.1831 | 28.78 | <0.001 |
|  | **Day 1 SIC Score** | 0.7777 | 0.8193 | 0.0416 | 5.35 | <0.001 |
| **MIMIC-IV 3.1** | **SOFA circulation** | 0.5752 | 0.933 | 0.3578 | 62.2 | <0.001 |
|  | **SOFA renal** | 0.5901 | 0.933 | 0.3429 | 58.12 | <0.001 |
|  | **Platelet Count** | 0.785 | 0.933 | 0.148 | 18.85 | <0.001 |
|  | **INR** | 0.8713 | 0.933 | 0.0617 | 7.08 | <0.001 |
|  | **SOFA respiratory** | 0.4831 | 0.933 | 0.4499 | 93.11 | <0.001 |
|  | **Lactate** | 0.6597 | 0.933 | 0.2733 | 41.43 | <0.001 |
|  | **Day 1 SIC Score** | 0.93 | 0.933 | 0.003 | 0.33 | 0.4908 |
| **eICU** | **SOFA circulation** | 0.569 | 0.8604 | 0.2914 | 51.2 | <0.001 |
|  | **SOFA renal** | 0.6057 | 0.8604 | 0.2548 | 42.06 | <0.001 |
|  | **Platelet Count** | 0.7388 | 0.8604 | 0.1216 | 16.46 | <0.001 |
|  | **INR** | 0.7403 | 0.8604 | 0.1202 | 16.23 | <0.001 |
|  | **SOFA respiratory** | 0.559 | 0.8604 | 0.3014 | 53.93 | <0.001 |
|  | **Lactate** | 0.6458 | 0.8604 | 0.2146 | 33.24 | <0.001 |
|  | **Day 1 SIC Score** | 0.8293 | 0.8604 | 0.0311 | 3.75 | <0.001 |
| **Zhongda Hospital** | **SOFA circulation** | 0.5969 | 0.822 | 0.2252 | 37.73 | <0.001 |
|  | **SOFA renal** | 0.6116 | 0.822 | 0.2104 | 34.4 | <0.001 |
|  | **Platelet Count** | 0.7627 | 0.822 | 0.0593 | 7.78 | <0.001 |
|  | **INR** | 0.6973 | 0.822 | 0.1248 | 17.89 | <0.001 |
|  | **SOFA respiratory** | 0.5142 | 0.822 | 0.3079 | 59.88 | <0.001 |
|  | **Lactate** | 0.6632 | 0.822 | 0.1588 | 23.95 | <0.001 |
|  | **Day 1 SIC Score** | 0.7823 | 0.822 | 0.0397 | 5.08 | <0.001 |

**Note:** ΔAUC represents the absolute increase in the Area Under the Receiver Operating Characteristic curve achieved by the Early-SIC model over the respective baseline feature. Relative Improvement (%) is calculated as (ΔAUC / Baseline AUC)*100%. All comparisons demonstrate the synergistic advantage of the machine learning approach over simplified linear or threshold-based scoring systems. **Abbreviations:** AUC, area under the curve; ΔAUC, change in AUC; SIC: Sepsis-induced coagulopathy; INR: International Normalized Ratio; SOFA: Sequential Organ Failure Assessment; MIMIC-IV: Medical Information Mart for Intensive Care IV; eICU: eICU Collaborative Research Database.

**Table S9. Performance of final model in predicting late-stage SIC onset (Days 4–7) in internal and external validation datasets.**

| **Variable** | **Internal Test Set** | **External Validation Set (MIMIC-IV 3.1)** | **External Validation Set (eICU)** | **External Validation Set (Zhongda Hospital)** |
| --- | --- | --- | --- | --- |
| **Accuracy** | 0.535 (0.513, 0.559) | 0.539 (0.514, 0.567) | 0.590 (0.564, 0.616) | 0.668 (0.637, 0.699) |
| **F1 Score** | 0.639 (0.616, 0.661) | 0.654 (0.628, 0.680) | 0.722 (0.700, 0.744) | 0.105 (0.063, 0.151) |
| **AUC** | 0.503 (0.475, 0.532) | 0.475 (0.440, 0.507) | 0.530 (0.481, 0.578) | 0.598 (0.515, 0.676) |
| **AUPRC** | 0.592 (0.560, 0.626) | 0.608 (0.575, 0.642) | 0.868 (0.842, 0.894) | 0.066 (0.045, 0.105) |
| **Sensitivity** | 0.701 (0.675, 0.728) | 0.692 (0.661, 0.727) | 0.616 (0.588, 0.643) | 0.404 (0.250, 0.550) |
| **Specificity** | 0.299 (0.269, 0.336) | 0.277 (0.240, 0.317) | 0.419 (0.346, 0.489) | 0.681 (0.650, 0.714) |
| **PPV** | 0.587 (0.560, 0.612) | 0.620 (0.589, 0.652) | 0.874 (0.851, 0.895) | 0.060 (0.036, 0.088) |
| **NPV** | 0.414 (0.373, 0.459) | 0.346 (0.301, 0.395) | 0.143 (0.114, 0.175) | 0.957 (0.938, 0.972) |

**Abbreviations:** SIC: Sepsis-induced coagulopathy; AUC: Area Under Curve; AUPRC: Area Under the Precision-Recall Curve; PPV: Positive Predictive Value; NPV: Negative Predictive Value; MIMIC-IV: Medical Information Mart for Intensive Care IV; eICU: eICU Collaborative Research Database

**Table S10. Quantitative assessment of Mean Risk Difference (MRD) in model outputs stratified by clinical subgroups across four datasets.**

| **Variable** | **Dataset** | **Non-exposed Group (n)** | **Mean Risk [95% CI]** | **Exposed Group (n)** | **Mean Risk [95% CI]** | ***p-value*** |
| --- | --- | --- | --- | --- | --- | --- |
| **Heparin Use** | MIMIC IV 2.2 | 6189 | 0.5266 [0.5197-0.5334] | 4844 | 0.5195 [0.5117-0.5272] | 0.1785 |
|  | MIMIC IV 3.0 | 599 | 0.5103 [0.4910-0.5296] | 772 | 0.5452 [0.5247-0.5657] | 0.0152 |
|  | eICU | 2561 | 0.6425 [0.6330-0.6520] | 287 | 0.6226 [0.5942-0.6510] | 0.1936 |
|  | ZhongDa Hospital | 148 | 0.5771 [0.5619-0.5924] | 1205 | 0.5885 [0.5288-0.6482] | 0.7169 |
| **Mechanical ventilation** | MIMIC IV 2.2 | 3736 | 0.5189 [0.5102-0.5275] | 7297 | 0.5259 [0.5195-0.5323] | 0.2023 |
|  | MIMIC IV 3.0 | 116 | 0.5330 [0.5072-0.5589] | 1255 | 0.5247 [0.5078-0.5415] | 0.5986 |
|  | eICU | 2041 | 0.6343 [0.6236-0.6450] | 807 | 0.6562 [0.6395-0.6728] | 0.0303 |
|  | ZhongDa Hospital | 652 | 0.5776 [0.5597-0.5954] | 701 | 0.5787 [0.5523-0.6051] | 0.9461 |
| **CRRT treatment** | MIMIC IV 2.2 | 10400 | 0.5229 [0.5177-0.5282] | 633 | 0.5317 [0.5099-0.5535] | 0.442 |
|  | MIMIC IV 3.0 | 1167 | 0.5272 [0.5124-0.5420] | 204 | 0.5259 [0.4795-0.5723] | 0.9583 |
|  | eICU | 2659 | 0.6400 [0.6306-0.6493] | 189 | 0.6476 [0.6139-0.6813] | 0.6706 |
|  | ZhongDa Hospital | 1124 | 0.5783 [0.5629-0.5938] | 229 | 0.5734 [0.5207-0.6262] | 0.8614 |
| **Hypertension** | MIMIC IV 2.2 | 4139 | 0.5287 [0.5201-0.5374] | 6894 | 0.5202 [0.5139-0.5266] | 0.1248 |
|  | MIMIC IV 3.0 | 580 | 0.5326 [0.5108-0.5544] | 791 | 0.5230 [0.5045-0.5415] | 0.5095 |
|  | eICU | 2652 | 0.6444 [0.6351-0.6537] | 196 | 0.5871 [0.5516-0.6227] | 0.0026 |
|  | ZhongDa Hospital | 617 | 0.5918 [0.5698-0.6137] | 736 | 0.5662 [0.5462-0.5862] | 0.0925 |
| **Diabetes** | MIMIC IV 2.2 | 7602 | 0.5205 [0.5143-0.5268] | 3431 | 0.5299 [0.5209-0.5389] | 0.0942 |
|  | MIMIC IV 3.0 | 885 | 0.5284 [0.5107-0.5461] | 486 | 0.5246 [0.5012-0.5481] | 0.8003 |
|  | eICU | 2785 | 0.6415 [0.6324-0.6506] | 63 | 0.5951 [0.5298-0.6604] | 0.1753 |
|  | ZhongDa Hospital | 924 | 0.5764 [0.5584-0.5944] | 429 | 0.5813 [0.5553-0.6073] | 0.7611 |
| **Chronic Kidney Disease** | MIMIC IV 2.2 | 8589 | 0.5007 [0.4949-0.5065] | 2444 | 0.6032 [0.5929-0.6136] | <0.0001 |
|  | MIMIC IV 3.0 | 1046 | 0.5014 [0.4853-0.5175] | 325 | 0.6096 [0.5823-0.6369] | <0.0001 |
|  | eICU | 2412 | 0.6332 [0.6233-0.6431] | 436 | 0.6807 [0.6596-0.7019] | 0.0001 |
|  | ZhongDa Hospital | 1234 | 0.5743 [0.5587-0.5898] | 119 | 0.6164 [0.5694-0.6634] | 0.099 |
| **Missing bilirubin data** | MIMIC IV 2.2 | 6763 | 0.5232 [0.5166-0.5299] | 4270 | 0.5237 [0.5156-0.5319] | 0.9258 |
|  | MIMIC IV 3.0 | 1371 | 0.5324 [0.5159-0.5490] | - | - | - |
|  | eICU | 2617 | 0.6413 [0.6320-0.6507] | 231 | 0.6308 [0.5978-0.6638] | 0.549 |
|  | ZhongDa Hospital | 1143 | 0.5829 [0.5673-0.5984] | 210 | 0.5227 [0.4759-0.5695] | 0.0174 |

**Note:** - indicates no data for this item.

**Abbreviations:** CI: Confidence interval; CRRT: Continuous Renal Replacement Therapy; MIMIC-IV: Medical Information Mart for Intensive Care IV; eICU: eICU Collaborative Research Database.

**Table S11. Sensitivity analysis of model performance: comparison of AUC between the full cohort and non-exposed cohorts across multiple clinical variables.**

| **Variable** | **Dataset** | **Full cohort AUC** | **Non-exposed cohort**  **AUC** | **ΔAUC** | ***p-value*** |
| --- | --- | --- | --- | --- | --- |
| **Heparin Use** | MIMIC-IV 2.2 | 0.8224 | 0.8220 | 0.0003 | 0.9024 |
|  | MIMIC-IV 3.1 | 0.9254 | 0.9269 | -0.0015 | 0.9052 |
|  | eICU | 0.8610 | 0.8634 | -0.0025 | 0.8383 |
|  | ZhongDa Hospital | 0.8273 | 0.8237 | 0.0036 | 0.8549 |
| **Mechanical ventilation** | MIMIC-IV 2.2 | 0.8224 | 0.8178 | 0.0045 | 0.5180 |
|  | MIMIC-IV 3.1 | 0.9254 | 0.9039 | 0.0215 | 0.2168 |
|  | eICU | 0.8610 | 0.8602 | 0.0007 | 0.9337 |
|  | ZhongDa Hospital | 0.8273 | 0.8157 | 0.0116 | 0.5811 |
| **CRRT treatment** | MIMIC-IV 2.2 | 0.8224 | 0.8229 | -0.0005 | 0.9343 |
|  | MIMIC-IV 3.1 | 0.9254 | 0.9244 | 0.0009 | 0.9157 |
|  | eICU | 0.8610 | 0.8618 | -0.0009 | 0.9443 |
|  | ZhongDa Hospital | 0.8273 | 0.8193 | 0.0080 | 0.6533 |
| **Hypertension** | MIMIC-IV 2.2 | 0.8224 | 0.8272 | -0.0048 | 0.5195 |
|  | MIMIC-IV 3.1 | 0.9254 | 0.9135 | 0.0119 | 0.4962 |
|  | eICU | 0.8610 | 0.8521 | 0.0089 | 0.7592 |
|  | ZhongDa Hospital | 0.8273 | 0.7720 | 0.0553 | 0.3972 |
| **Diabetes** | MIMIC-IV 2.2 | 0.8224 | 0.8360 | -0.0137 | 0.0598 |
|  | MIMIC-IV 3.1 | 0.9254 | 0.9328 | -0.0074 | 0.5546 |
|  | eICU | 0.8610 | 0.8602 | 0.0007 | 0.9640 |
|  | ZhongDa Hospital | 0.8273 | 0.8437 | -0.0164 | 0.4191 |
| **CKD** | MIMIC-IV 2.2 | 0.8224 | 0.8265 | -0.0042 | 0.4751 |
|  | MIMIC-IV 3.1 | 0.9254 | 0.9238 | 0.0015 | 0.8842 |
|  | eICU | 0.8610 | 0.8601 | 0.0009 | 0.8971 |
|  | ZhongDa Hospital | 0.8273 | 0.8429 | -0.0155 | 0.3845 |
| **Missing bilirubin data** | MIMIC-IV 2.2 | 0.8224 | 0.8185 | 0.0038 | 0.5013 |
|  | MIMIC-IV 3.1 | 0.9254 | 0.9196 | 0.0057 | 0.6526 |
|  | eICU | 0.8610 | 0.8639 | -0.0029 | 0.7728 |
|  | ZhongDa Hospital | 0.8273 | 0.8357 | -0.0084 | 0.6401 |

**Abbreviations:** CRRT: Continuous Renal Replacement Therapy; CKD: Chronic Kidney Disease; AUC: Area Under Curve; MIMIC-IV: Medical Information Mart for Intensive Care IV; eICU: eICU Collaborative Research Database.

**Table S12. Subgroup analysis of model discriminative performance stratified by therapeutic interventions, medical history, and baseline organ dysfunction across four cohorts.**

| **Dataset** | **Group** | **AUC** | **ΔAUC** | **AUPRC** | **Sensitivity** | **Specificity** | **PPV** | **NPV** | **F1 Score** |
| --- | --- | --- | --- | --- | --- | --- | --- | --- | --- |
| **MIMIC-IV 2.2** | Non-Heparin | 0.822 | 0.0006 | 0.8545 | 0.8843 | 0.5125 | 0.683 | 0.7802 | 0.7707 |
|  | Heparin | 0.8226 |  | 0.8497 | 0.7984 | 0.6812 | 0.7452 | 0.7435 | 0.7709 |
| **MIMIC-IV 3.1** | Non-Heparin | 0.9269 | -0.004 | 0.9488 | 0.8834 | 0.8257 | 0.8568 | 0.8571 | 0.8699 |
|  | Heparin | 0.9229 |  | 0.9555 | 0.8822 | 0.7954 | 0.8691 | 0.8082 | 0.8756 |
| **eICU** | Non-Heparin | 0.8634 | -0.0257 | 0.9433 | 0.9541 | 0.4221 | 0.8183 | 0.7608 | 0.881 |
|  | Heparin | 0.8377 |  | 0.9356 | 0.9953 | 0.2192 | 0.7889 | 0.9556 | 0.8802 |
| **Zhongda Hospital** | Non-Heparin | 0.8237 | 0.0306 | 0.5795 | 0.8072 | 0.7153 | 0.497 | 0.9162 | 0.6152 |
|  | Heparin | 0.8543 |  | 0.6959 | 0.9355 | 0.7333 | 0.6444 | 0.9569 | 0.7632 |
| **MIMIC-IV 2.2** | Non-MV | 0.8178 | 0.0070 | 0.8496 | 0.7150 | 0.7621 | 0.7800 | 0.6938 | 0.7461 |
|  | MV | 0.8248 |  | 0.8540 | 0.7337 | 0.7574 | 0.7809 | 0.7070 | 0.7566 |
| **MIMIC-IV 3.1** | Non-MV | 0.9039 | 0.0300 | 0.9444 | 0.7689 | 0.8824 | 0.9104 | 0.7105 | 0.8337 |
|  | MV | 0.9338 |  | 0.9559 | 0.8172 | 0.9053 | 0.9160 | 0.7967 | 0.8638 |
| **eICU** | Non-MV | 0.8602 | 0.0015 | 0.9395 | 0.8364 | 0.6901 | 0.8750 | 0.6193 | 0.8553 |
|  | MV | 0.8617 |  | 0.9498 | 0.8537 | 0.6875 | 0.8974 | 0.5946 | 0.8750 |
| **Zhongda Hospital** | Non-MV | 0.8157 | 0.0338 | 0.5809 | 0.9099 | 0.5432 | 0.4097 | 0.9454 | 0.5650 |
|  | MV | 0.8495 |  | 0.6242 | 0.9391 | 0.5714 | 0.4557 | 0.9609 | 0.6136 |
| **MIMIC-IV 2.2** | Non-CRRT | 0.8229 | -0.0083 | 0.8514 | 0.7283 | 0.7593 | 0.7793 | 0.7054 | 0.7529 |
|  | CRRT | 0.8146 |  | 0.8701 | 0.7091 | 0.7549 | 0.8025 | 0.6488 | 0.7529 |
| **MIMIC-IV 3.1** | Non-CRRT | 0.9244 | 0.0080 | 0.9524 | 0.7975 | 0.8971 | 0.9140 | 0.7636 | 0.8517 |
|  | CRRT | 0.9324 |  | 0.9518 | 0.8481 | 0.9155 | 0.9178 | 0.8442 | 0.8816 |
| **eICU** | Non-CRRT | 0.8618 | -0.0125 | 0.9431 | 0.8409 | 0.6915 | 0.8821 | 0.6130 | 0.8610 |
|  | CRRT | 0.8494 |  | 0.9349 | 0.8489 | 0.6600 | 0.8741 | 0.6111 | 0.8613 |
| **ZDYY** | Non-CRRT | 0.8193 | 0.0933 | 0.5764 | 0.9126 | 0.5464 | 0.4190 | 0.9458 | 0.5743 |
|  | CRRT | 0.9126 |  | 0.7794 | 1.0000 | 0.6184 | 0.4912 | 1.0000 | 0.6588 |
| **MIMIC-IV 2.2** | Non-Hypertension | 0.8360 | -0.0221 | 0.8678 | 0.7388 | 0.7773 | 0.8041 | 0.7063 | 0.7700 |
|  | Hypertension | 0.8139 |  | 0.8425 | 0.7199 | 0.7485 | 0.7663 | 0.6999 | 0.7424 |
| **MIMIC-IV 3.1** | Non-Hypertension | 0.9328 | -0.0123 | 0.9539 | 0.8154 | 0.8902 | 0.9044 | 0.7909 | 0.8576 |
|  | Hypertension | 0.9205 |  | 0.9512 | 0.7935 | 0.9063 | 0.9217 | 0.7595 | 0.8528 |
| **eICU** | Non-Hypertension | 0.8602 | 0.0074 | 0.9431 | 0.8438 | 0.6867 | 0.8827 | 0.6115 | 0.8628 |
|  | Hypertension | 0.8676 |  | 0.9372 | 0.8074 | 0.7213 | 0.8651 | 0.6286 | 0.8352 |
| **Zhongda Hospital** | Non-Hypertension | 0.8437 | -0.0332 | 0.7000 | 0.9180 | 0.5721 | 0.4941 | 0.9388 | 0.6424 |
|  | Hypertension | 0.8105 |  | 0.4871 | 0.9221 | 0.5373 | 0.3641 | 0.9600 | 0.5221 |
| **MIMIC-IV 2.2** | Non-Diabetes | 0.8265 | -0.0138 | 0.8527 | 0.7324 | 0.7657 | 0.7814 | 0.7145 | 0.7561 |
|  | Diabetes | 0.8127 |  | 0.8522 | 0.7159 | 0.7434 | 0.7790 | 0.6744 | 0.7461 |
| **MIMIC-IV 3.1** | Non-Hypertension | 0.9238 | 0.0057 | 0.9526 | 0.7949 | 0.9062 | 0.9208 | 0.7630 | 0.8532 |
|  | Diabetes | 0.9295 |  | 0.9522 | 0.8168 | 0.8873 | 0.9028 | 0.7908 | 0.8577 |
| **eICU** | Non-Hypertension | 0.8601 | 0.0313 | 0.9428 | 0.8418 | 0.6879 | 0.8823 | 0.6101 | 0.8616 |
|  | Diabetes | 0.8913 |  | 0.9297 | 0.8250 | 0.7391 | 0.8462 | 0.7083 | 0.8354 |
| **Zhongda Hospital** | Non-Hypertension | 0.8429 | -0.0503 | 0.6030 | 0.9389 | 0.5557 | 0.4283 | 0.9625 | 0.5882 |
|  | Diabetes | 0.7925 |  | 0.5772 | 0.8796 | 0.5445 | 0.4167 | 0.9244 | 0.5655 |
| **MIMIC-IV 2.2** | Non-CKD | 0.8265 | -0.0138 | 0.8527 | 0.7324 | 0.7657 | 0.7814 | 0.7145 | 0.7561 |
|  | CKD | 0.8127 |  | 0.8522 | 0.7159 | 0.7434 | 0.7790 | 0.6744 | 0.7461 |
| **MIMIC-IV 3.1** | Non-CKD | 0.9238 | 0.0057 | 0.9526 | 0.7949 | 0.9062 | 0.9208 | 0.7630 | 0.8532 |
|  | CKD | 0.9295 |  | 0.9522 | 0.8168 | 0.8873 | 0.9028 | 0.7908 | 0.8577 |
| **eICU** | Non-CKD | 0.8601 | 0.0313 | 0.9428 | 0.8418 | 0.6879 | 0.8823 | 0.6101 | 0.8616 |
|  | CKD | 0.8913 |  | 0.9297 | 0.8250 | 0.7391 | 0.8462 | 0.7083 | 0.8354 |
| **Zhongda Hospital** | Non-CKD | 0.8429 | -0.0503 | 0.6030 | 0.9389 | 0.5557 | 0.4283 | 0.9625 | 0.5882 |
|  | CKD | 0.7925 |  | 0.5772 | 0.8796 | 0.5445 | 0.4167 | 0.9244 | 0.5655 |
| **MIMIC-IV 2.2** | Missing bilirubin data | 0.8272 | -0.008 | 0.8566 | 0.731 | 0.758 | 0.7785 | 0.7078 | 0.754 |
|  | Non-missing data | 0.8192 |  | 0.8498 | 0.7246 | 0.7597 | 0.782 | 0.6987 | 0.7522 |
| **MIMIC-IV 3.1** | Missing bilirubin data | 0.9135 | 0.0171 | 0.947 | 0.7736 | 0.9236 | 0.9318 | 0.7513 | 0.8454 |
|  | Non-missing data | 0.9305 |  | 0.9547 | 0.8133 | 0.8904 | 0.9084 | 0.7812 | 0.8582 |
| **eICU** | Missing bilirubin data | 0.8521 | 0.0094 | 0.9351 | 0.8084 | 0.7031 | 0.8766 | 0.5844 | 0.8411 |
|  | Non-missing data | 0.8615 |  | 0.9431 | 0.8444 | 0.6882 | 0.882 | 0.6157 | 0.8628 |
| **Zhongda Hospital** | Missing bilirubin data | 0.772 | 0.0573 | 0.4409 | 0.8889 | 0.6437 | 0.3404 | 0.9655 | 0.4923 |
|  | Non- missing data | 0.8293 |  | 0.6035 | 0.9216 | 0.5429 | 0.4305 | 0.9487 | 0.5868 |

**Abbreviations:** MV: Machine Ventilation; CRRT: Continuous Renal Replacement Therapy; CKD: Chronic Kidney Disease; AUC: Area Under Curve; AUPRC: Area Under the Precision-Recall Curve; PPV: Positive Predictive Value; NPV: Negative Predictive Value; MIMIC-IV: Medical Information Mart for Intensive Care IV; eICU: eICU Collaborative Research Database.

**Table S13. Lead time analysis of the Early-SIC model performance after excluding early-onset cases (Day 1 SIC).**

| **Dataset** | **Exclusion Rate (%)** | **Positive Rate (%)** | **Accuracy** | **F1 Score** | **AUC** | **AUPRC** | **Sensitivity** | **Specificity** | **PPV** | **NPV** |
| --- | --- | --- | --- | --- | --- | --- | --- | --- | --- | --- |
| **Derivation cohort**  **（MIMIC-IV 2.2）** | 46.46 | 32.33 | 0.805 | 0.726 | 0.864 | 0.742 | 0.799 | 0.808 | 0.665 | 0.894 |
| **MIMIC-IV 3.1** | 72.08 | 64.23 | 0.516 | 0.588 | 0.517 | 0.664 | 0.538 | 0.477 | 0.649 | 0.365 |
| **eICU** | 72.82 | 35.53 | 0.571 | 0.339 | 0.562 | 0.417 | 0.309 | 0.715 | 0.374 | 0.653 |
| **Zhongda Hospital** | 57.02 | 7.30 | 0.834 | 0.195 | 0.706 | 0.132 | 0.275 | 0.878 | 0.151 | 0.939 |
| **Average** | 62.10 | 34.80 | 0.6815 | 0.462 | 0.662 | 0.489 | 0.48 | 0.72 | 0.46 | 0.713 |

**Abbreviations:** SIC: Sepsis-induced coagulopathy; AUC: Area Under Curve; AUPRC: Area Under the Precision-Recall Curve; PPV: Positive Predictive Value; NPV: Negative Predictive Value; MIMIC-IV: Medical Information Mart for Intensive Care IV; eICU: eICU Collaborative Research Database.

**Table S14. Predictive thresholds and performance of the final model across different datasets.**

| **Dataset** | **Optimal_threshold** | **Youden_index** | **AUC** | **Sensitivity** | **Specificity** | **Accuracy** | **PPV** | **NPV** |
| --- | --- | --- | --- | --- | --- | --- | --- | --- |
| **Training set** | 0.56 | 0.50 | 0.83 | 0.73 | 0.77 | 0.75 | 0.76 | 0.74 |
| **Internal validation set** | 0.54 | 0.50 | 0.83 | 0.74 | 0.76 | 0.75 | 0.79 | 0.71 |
| **Internal test set** | 0.63 | 0.50 | 0.82 | 0.68 | 0.83 | 0.75 | 0.81 | 0.70 |
| **External validation set**  **(MIMIC-IV 3.1)** | 0.51 | 0.71 | 0.93 | 0.85 | 0.86 | 0.86 | 0.90 | 0.81 |
| **External validation set**  **(eICU)** | 0.61 | 0.55 | 0.86 | 0.80 | 0.75 | 0.79 | 0.90 | 0.58 |
| **External validation set**  **(Zhongda Hospital)** | 0.62 | 0.52 | 0.83 | 0.88 | 0.64 | 0.71 | 0.47 | 0.94 |

**Abbreviations:** AUC: Area Under Curve; PPV: Positive Predictive Value; NPV: Negative Predictive Value; MIMIC-IV: Medical Information Mart for Intensive Care IV; eICU: eICU Collaborative Research Database.





**Figure S1. Density Distribution Comparison Before and After Imputation in the Derivation Cohort.**

CKD: Chronic Kidney Disease; CRRT: Continuous Renal Replacement Therapy; BMI: Body Mass Index; sofa: Sequential Organ Failure Assessment; Hb: Hemoglobin; MCH: Mean Corpuscular Hemoglobin; WBC: White Blood Cell; INR: International Normalized Ratio; PT: Prothrombin Time; PTT: Partial Thromboplastin Time; BUN: Blood Urea Nitrogen; PH: potential of hydrogen; PaO_2_: Partial Pressure of Oxygen; P/F Ratio: PaO_2_/FiO_2_ Ratio; MAP: Mean Arterial Pressure.





**Figure S2. The weights of each feature incorporated in different feature selection methods.**

Four feature selection methods were employed: the Kruskal-Wallis test (A), RFECV nested with a tree model (Random Forest) (B), RFECV nested with a linear model (Logistic Regression) (C), and Null Importance (D and E). Null Importance feature selection uses permutation tests to assess the significance of actual feature importance against the distribution of feature importance when fitted to noise (shuffled target) through information gain and splitting in the tree model. RFECV: Recursive Feature Elimination with Cross-Validation; RF: Random Forest; LR: Logistic Regression; INR: International Normalized Ratio; PT: Prothrombin Time; PTT: Partial Thromboplastin Time; sofa: Sequential Organ Failure Assessment; BUN: Blood Urea Nitrogen; Hb: Hemoglobin; MAP: Mean Arterial Pressure; WBC: White Blood Cell; P/F Ratio: PaO_2_/FiO_2_ Ratio; PaO_2_:Partial Pressure of Oxygen; CKD: Chronic Kidney Disease; ICU: Intensive Care Unit; BMI: Body Mass Index; CRRT: Continuous Renal Replacement Therapy; PH: potential of hydrogen; MCH: Mean Corpuscular Hemoglobin. Hb: Hemoglobin.


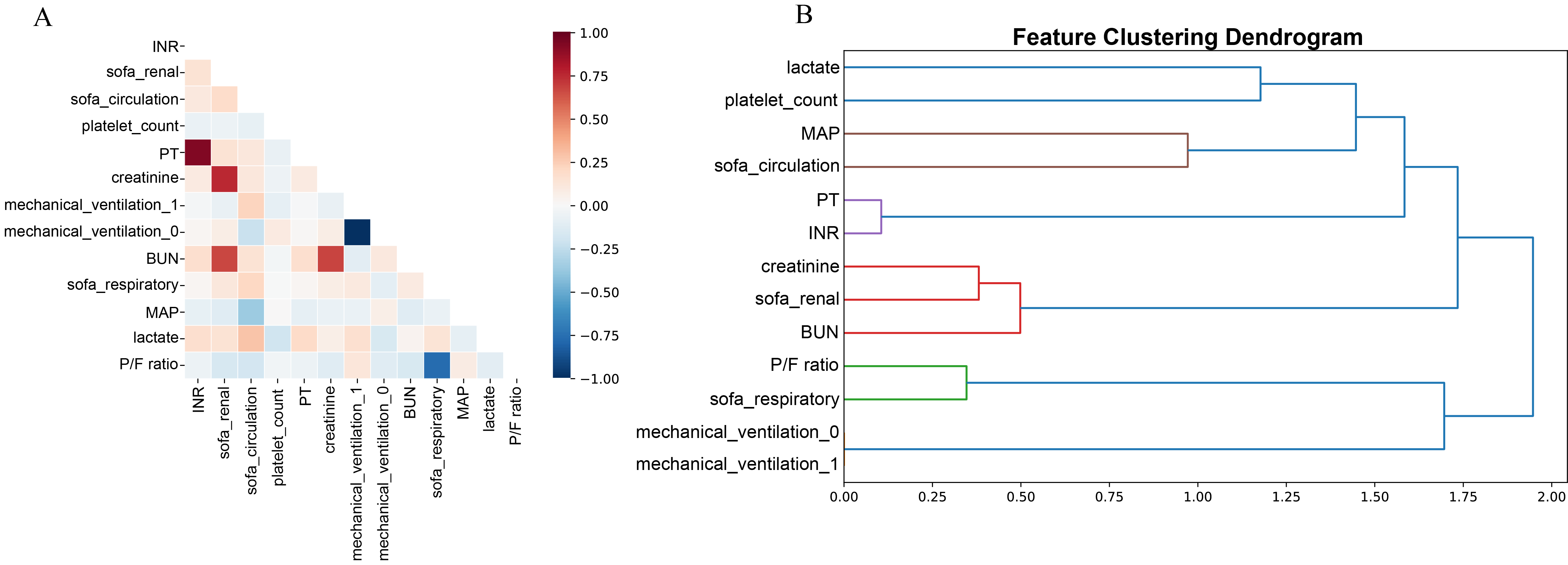


**Figure S3. Collinearity analysis in feature selection.**

Heatmap of correlation analysis based on Pearson covariance among candidate features(A); Feature clustering dendrogram(B). INR: International Normalized Ratio; sofa: Sequential Organ Failure Assessment; PT: Prothrombin Time; BUN: Blood Urea Nitrogen; MAP: Mean Arterial Pressure; P/F Ratio: PaO2/FiO2 Ratio.


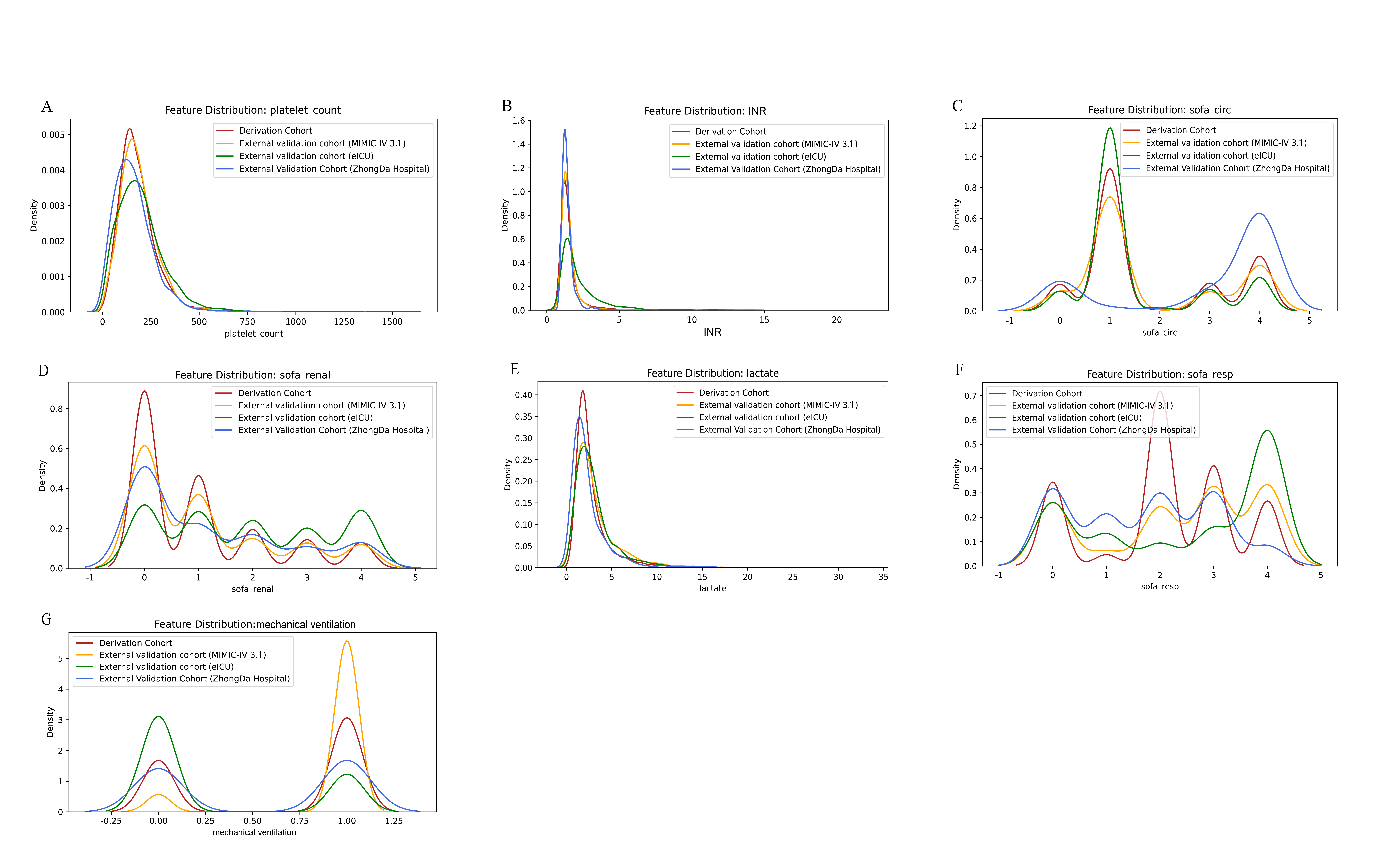


**Figure S4. Comparison of density distributions for selected clinical features between the Derivation set and External validation set.**

This figure illustrates the probability density distributions of platelet count (A), INR (B), circulatory SOFA score (C), renal SOFA score (D), lactate level (E), respiratory SOFA score (F), and mechanical ventilation status (G) in the Derivation set (red), MIMIC-IV 3.1 external validation set (orange), eICU external validation set(green), and ZhongDa Hospital external validation set (blue). INR: International Normalized Ratio; sofa: Sequential Organ Failure Assessment; MIMIC-IV: Medical Information Mart for Intensive Care IV; eICU: eICU Collaborative Research Database.


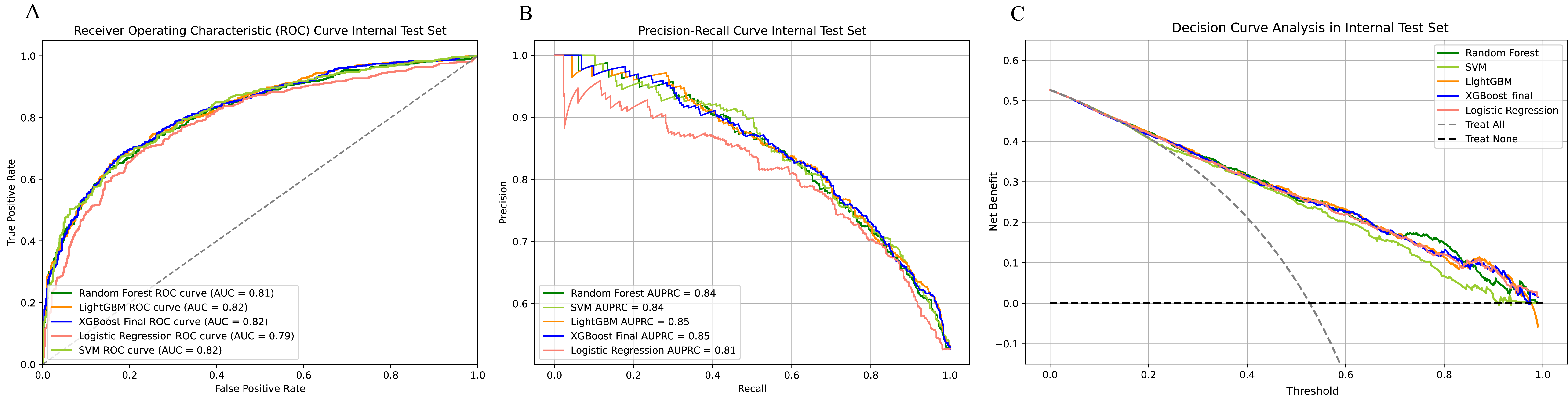


**Figure S5. The performance and comparison of five different predictive models in internal test set.**

(A) ROC curves for the internal test set; (B) Precision-recall curves for the internal test set; (C) Decision curve analysis for the internal test set. ROC: Receiver Operating Characteristic; AUC: Area Under Curve; XGBoost: Extreme Gradient Boosting; LightGBM: Light Gradient Boosting Machine; SVM: Support Vector Machine; AUPRC: Area Under the Precision-Recall Curve


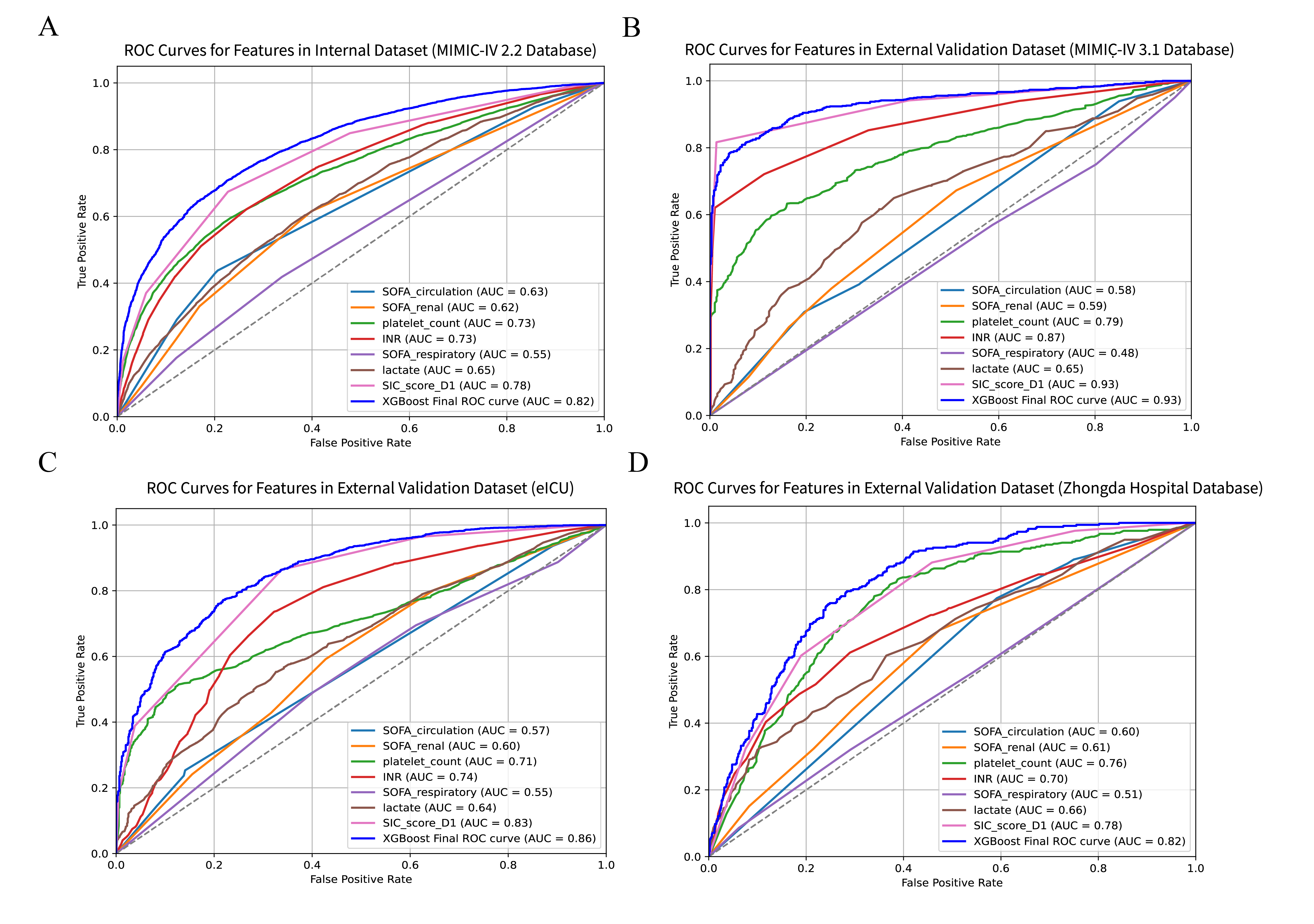
**Figure S6. Comparison of Receiver Operating Characteristic (ROC) Curves Based on the Predicted Scores of the XGBoost Model and Benchmark Clinical Indicators or Scores.**

(A) The performance in internal set (from MIMIC-IV 2.2 dataset); (B) Performance in the MIMIC-IV 3.1 external validation set; (C)Performance in the eICU external validation set; (D) Performance in the Zhongda Hospital external validation set.

ROC: Receiver Operating Characteristic; AUC: Area Under Curve; XGBoost: Extreme Gradient Boosting; SIC: Sepsis-induced coagulopathy; INR: International Normalized Ratio; SOFA: Sequential Organ Failure Assessment; MIMIC-IV: Medical Information Mart for Intensive Care IV; eICU: eICU Collaborative Research Databae.


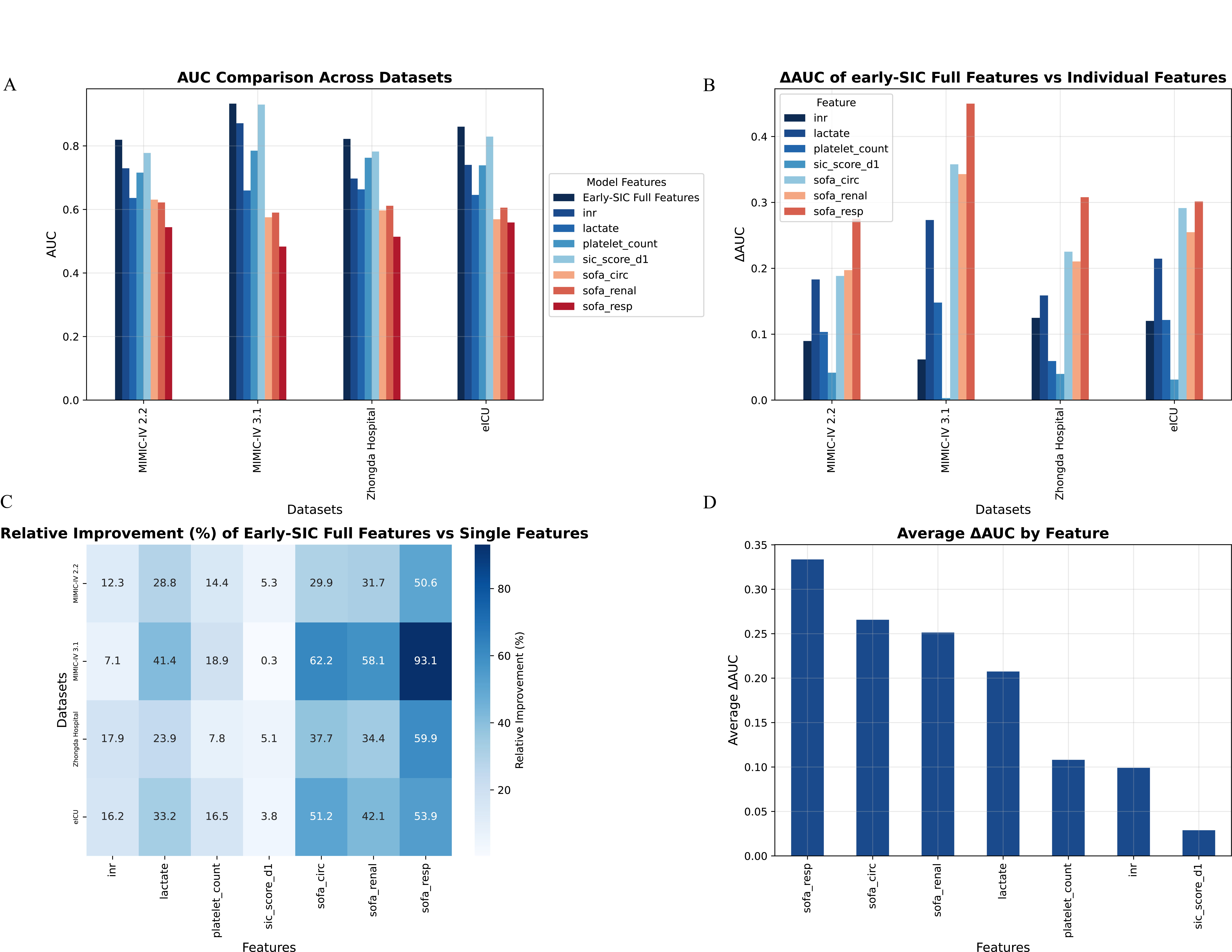


**Figure S7. Comprehensive performance evaluation and incremental value analysis of the Early-SIC model across multiple datasets.**

(A) AUC Comparison Across Datasets; (B) ΔAUC of Early-SIC Full Features vs. Individual Features; (C) Relative Improvement (%) Heatmap; (D) Average ΔAUC by Features. AUC, area under the curve; ΔAUC, change in AUC; SIC: Sepsis-induced coagulopathy; INR: International Normalized Ratio; SOFA: Sequential Organ Failure Assessment; MIMIC-IV: Medical Information Mart for Intensive Care IV; eICU: eICU Collaborative Research Database.


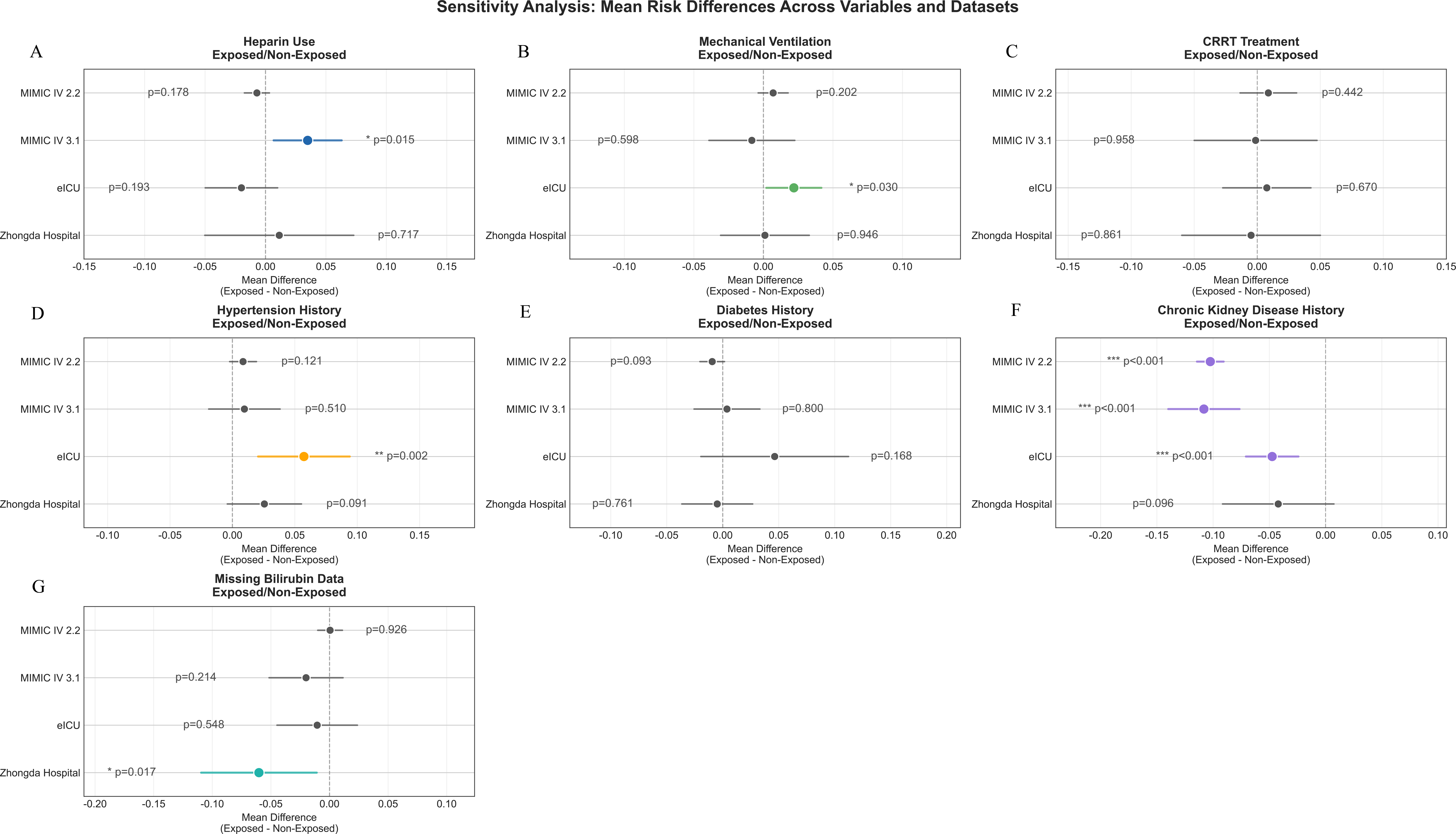


**Figure S8. Sensitivity analysis of model-predicted risk scores using Mean Risk Difference (MRD) across therapeutic interventions, medical history, and data missingness patterns.** (A) Heparin Use;(B) Mechanical Ventilation;(C) CRRT Treatment;(D) Hypertension;(E) Diabetes;(F) Chronic Kidney Disease;(G) Data Missingness (Bilirubin).



 **Figure S9. Proposed clinical integration framework and actionable management pathway for early sepsis-induced coagulopathy (SIC) prediction.**

**NOTE:** The flowchart delineates the systematic integration of the XGBoost-based Early-SIC model into clinical workflows to facilitate early identification and proactive management of sepsis patients. Model Implementation: Upon ICU admission, routine clinical data are collected within the first 24 hours. The XGBoost model executes risk prediction at the 24-hour mark post-admission. Based on the optimized performance metrics, a predicted probability of ≥54% is utilized as the clinical decision threshold for a "High-Risk Alert". Actionable Pathway: Patients flagged as high-risk enter a tiered management protocol designed to mitigate coagulopathy progression:

Tier 1 (Enhanced Monitoring): Focused on frequent reassessment of coagulation markers (e.g., PT, APTT, D-dimer every 6-12 hours) and point-of-care testing (TEG/ROTEM).

Tier 2 (Proactive Management): Includes ruling out reversible causes and optimizing prophylactic anticoagulation or blood product transfusion strategies.

Tier 3 (Multi-disciplinary Consultation and Advanced Intervention): Involves joint ICU and Hematology consultation for definitive therapy and source control escalation.

Standard Care: Patients with a predicted risk < 54% continue with routine sepsis monitoring and standard care protocols. This structured approach aims to bridge the gap between machine learning-based risk assessment and real-time clinical intervention.

ICU: Intensive Care Unit; SIC: Sepsis-induced Coagulopathy; PT: Prothrombin Time; APTT: Activated Partial Thromboplastin Time; INR: International Normalized Ratio; FIB: Fibrinogen; XGBoost: Extreme Gradient Boosting; TEG: Thromboelastography; ROTEM: Rotational Thromboelastometry; DIC: Disseminated Intravascular Coagulation; MDT: Multidisciplinary Team.
